# Characterization and quantification of the fungal microbiome in serial samples from individuals with cystic fibrosis

**DOI:** 10.1186/2049-2618-2-40

**Published:** 2014-11-03

**Authors:** Sven D Willger, Sharon L Grim, Emily L Dolben, Anna Shipunova, Thomas H Hampton, Hilary G Morrison, Laura M Filkins, George A O‘Toole, Lisa A Moulton, Alix Ashare, Mitchell L Sogin, Deborah A Hogan

**Affiliations:** 1 Geisel School of Medicine at Dartmouth, Hanover, NH, USA; 2 Josephine Bay Paul Center for Comparative Molecular Biology and Evolution, Marine Biological Laboratory, Woods Hole, MA, USA; 3 Dartmouth-Hitchcock Medical Center, Section of Pulmonary and Critical Care Medicine, Lebanon, NH, USA; 4 Earth and Environmental Sciences, University of Michigan, Ann Arbor, MI, USA

## Abstract

**Background:**

Human-associated microbial communities include fungi, but we understand little about which fungal species are present, their relative and absolute abundances, and how antimicrobial therapy impacts fungal communities. The disease cystic fibrosis (CF) often involves chronic airway colonization by bacteria and fungi, and these infections cause irreversible lung damage. Fungi are detected more frequently in CF sputum samples upon initiation of antimicrobial therapy, and several studies have implicated the detection of fungi in sputum with worse outcomes. Thus, a more complete understanding of fungi in CF is required.

**Results:**

We characterized the fungi and bacteria in expectorated sputa from six CF subjects. Samples were collected upon admission for systemic antibacterial therapy and upon the completion of treatment and analyzed using a pyrosequencing-based analysis of fungal internal transcribed spacer 1 (ITS1) and bacterial 16S rDNA sequences. A mixture of *Candida* species and *Malassezia* dominated the mycobiome in all samples (74%–99% of fungal reads). There was not a striking trend correlating fungal and bacterial richness, and richness showed a decline after antibiotic therapy particularly for the bacteria. The fungal communities within a sputum sample resembled other samples from that subject despite the aggressive antibacterial therapy. Quantitative PCR analysis of fungal 18S rDNA sequences to assess fungal burden showed variation in fungal density in sputum before and after antibacterial therapy but no consistent directional trend. Analysis of *Candida* ITS1 sequences amplified from sputum or pure culture-derived genomic DNA from individual *Candida* species found little (<0.5%) or no variation in ITS1 sequences within or between strains, thereby validating this locus for the purpose of *Candida* species identification. We also report the enhancement of the publically available Visualization and Analysis of Microbial Population Structures (VAMPS) tool for the analysis of fungal communities in clinical samples.

**Conclusions:**

Fungi are present in CF respiratory sputum. In CF, the use of intravenous antibiotic therapy often does not profoundly impact bacterial community structure, and we observed a similar stability in fungal species composition. Further studies are required to predict the effects of antibacterials on fungal burden in CF and fungal community stability in non-CF populations.

## Background

Subjects with the genetic disease cystic fibrosis (CF) often have complex, chronic mixed bacterial-fungal lung infections that cause severe lung damage that can progress to respiratory failure. Molecular profiling studies strongly support a model in which CF lung infections contain multiple bacteria [1-3], and recent work has revealed that these communities are often surprisingly stable within subjects over time [2,4-7]. Fungal species, such as *Candida albicans* and *Aspergillus fumigatus*, are also commonly detected in sputum cultures. Culture-based studies have detected *C. albicans* in ~40%–75% CF subjects [8-21] and described its association with reduced lung function [16,22]. A large European study found that the presence of fungi by laboratory culture correlated with a 5%–10% lower lung function in CF [20]. Two prospective studies have linked the detection of either *C. albicans* or *A. fumigatus* to lower lung function and increased frequency of disease exacerbations [16,22]. Other fungi have also been detected in the CF lung [19,23-27]. Challenges associated with the culturing of fungi, due to slow growth, specific medium requirements [28], and the lack of quantitative methods due in part to filamentous morphologies [29], have made it difficult to describe fungal species and their relative burdens in the CF lung. Consequently, despite data that suggest that the mycobiome poses a health threat to individuals with CF [16,22], there is not yet a comprehensive standard of care upon the detection of fungi in sputum.

Individuals with CF-associated lung infections usually experience periods of stable lung function interspersed with episodes of disease exacerbation, which is loosely defined as a period of impaired lung function or the worsening of other health metrics that requires hospitalization for the administration of one or more intravenous antibiotics. The effects of antibiotic treatment on bacterial numbers and community structure are not fully understood and variable across subjects. One recent study found that antibiotic therapy does not cause reproducible shifts in bacterial community structure or bacterial density within sputum [30], while another study reported that antibiotic use was associated with decreased diversity and short-term shifts in community structure [4]. The effects of antibiotic therapy on fungal loads or fungal species composition in the lung are not well known. Several studies have indicated that antibiotic usage precedes the increased detection of fungi in sputum [31-36]. Furthermore, antibiotic therapy in other systems correlates with an increased incidence of fungal infections, such as bloodstream infections [37-40], oral candidosis [41], and vaginal candidiasis [42], and increased fungal loads in the gastrointestinal tract [43-45]. A full characterization of the fungal species within sputum and other host-associated microbial communities will require the community-wide implementation of lysis methods that are effective for all bacterial and fungal species and the use of a combination of molecular methods that provide information on all of the species present. Most molecular analyses of host-associated fungi have relied upon amplification and sequencing of the internal transcribed spacer 1 (ITS1) region within the ribosomal RNA operon [46-49]. Due to a high degree of variation between even closely related species [50-52], this region can serve as a unique taxonomic identifier in both traditional capillary and next generation DNA sequencing studies [46-49].

In this report, we assessed the fungi within the CF lung in subjects hospitalized for treatment with antibacterial antibiotics and advanced the development of tools for the analysis of the fungal component of the microbiome (mycobiome) within clinical samples. Using primers that amplify the ITS1 region, we characterized the mycobiome and bacterial microbiome in expectorated sputum samples from six subjects before and after antibiotic therapy. *Candida* species dominated the mycobiomes, and their relative abundances were stable over time in contrast to less stable bacterial community structures. Using these samples, we also quantified the numbers of fungal genomes within each sample and found variability in how fungal densities in sputum changed over time and after treatment with antibacterial drugs. Lastly, towards facilitating future mycobiome studies, we assess intra-strain variability in *Candida* ITS1 sequences and describe improvement of the fungal ITS1 reference database associated with VAMPS (Visualization and Analysis of Microbial Population Structures) [53] and its use in analyses of fungal ITS1 sequences amplified from host-associated microbial communities.

## Results

### Adapting a publicly available tool for mycobiome analysis

To aid the analysis of fungal ITS1 sequences, particularly those amplified from clinical samples, we improved the ITS reference database for Global Alignment for Sequence Taxonomy (GAST) [54] analyses within VAMPS, a widely used web-based tool [53] that resolves phylogenetic affinity of marker gene sequences from diverse bacterial communities ( [2,55-57] as examples). The reference database for assigning the taxonomic affinity of fungal ITS1 amplicon sequences derives from the UNITE database [58]. Our modifications included a number of corrections to the taxonomic information within records for medically relevant fungi, and the removal of some sequences that were exact matches to well-characterized plant sequences that had been incorrectly annotated as being of fungal origin. We also increased representation of the human-associated fungi *Candida* and *Malassezia* in the database by adding all Genbank database entries that contained the keywords “ITS”, “*Candida*”, and “*Malassezia*” and had complete annotations. This step increased the number of *Candida* species from 251 to 284 and the number of unique accession IDs containing the keyword “*Candida*” from 930 to 2021. For *Malassezia*, 72 unique accession IDs containing the keyword “*Malassezia”* from Genbank with complete genus and species information were added.

### Overall assessment of the fungi identified in sputum from inpatient subjects

Using VAMPS, we analyzed the fungal community in CF sputum from six hospitalized subjects that received subject-specific antibacterial treatments (Additional file 1: Table S1). Using primers that match conserved flanking sequences, we PCR-amplified the fungal ITS1 sequences from sputum DNA samples for pyrosequencing analyses. We determined the reproducibility of the fungal ITS1 amplification and deep sequencing protocol by analyzing technical replicate libraries created from the same DNA that had been extracted from a de-identified sputum sample collected for the purposes of the method development. The results from the two technical replicates were very similar in terms of the fraction of reads assigned to each fungus (<1% difference in percent abundance per sample) (Additional file 2: Figure S1).

**Figure 1:**
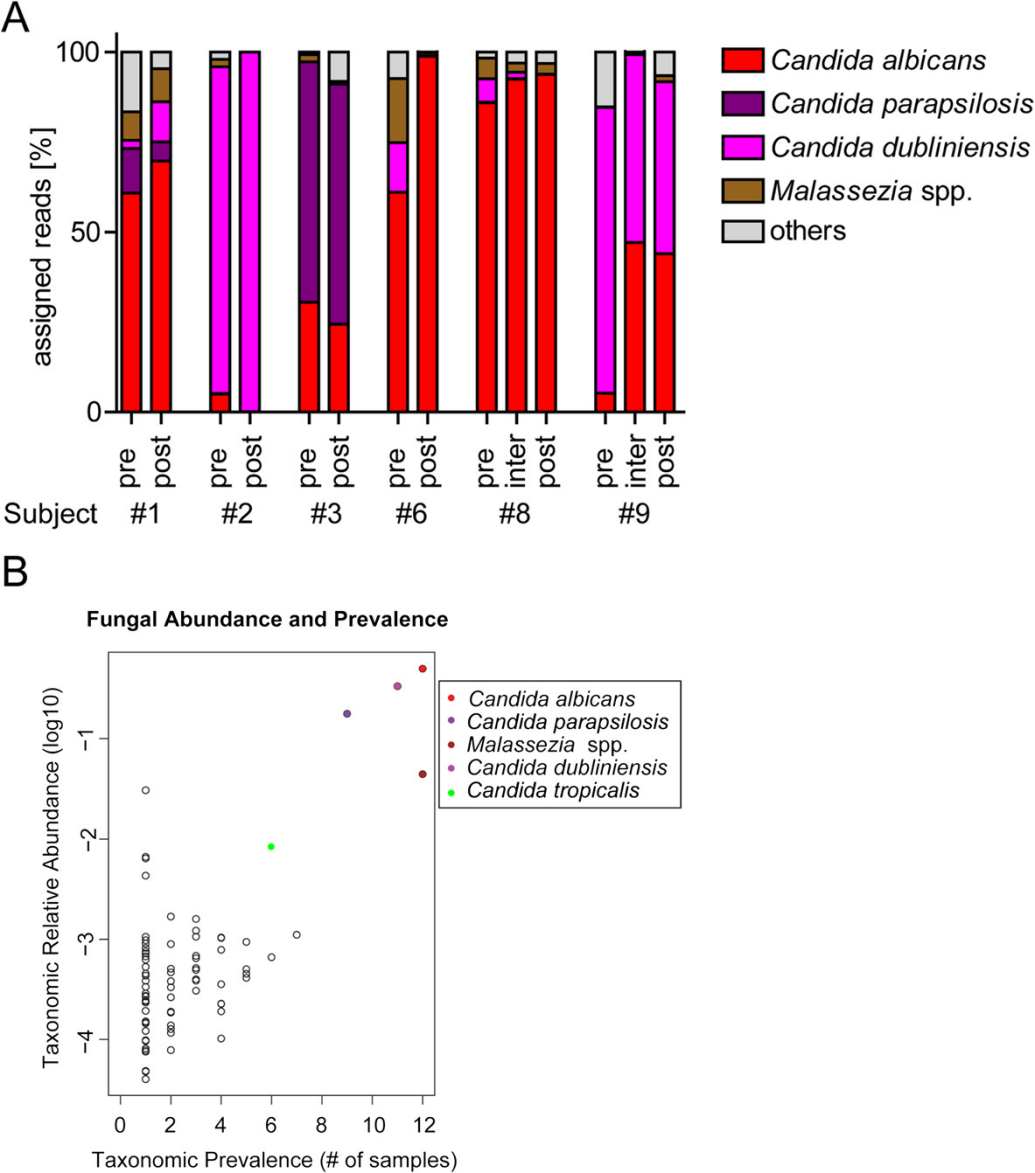
**Characterization of the fungal communities of sputum samples from CF subjects. (A)** Fraction of pyrosequencing reads assigned to each of the top four fungal taxa detected in CF subjects immediately after an exacerbation and before starting an antibacterial therapy (pre) and approximately 2 weeks afterwards (post) or while hospitalized (inter). The legend indicates the color assigned to each indicated fungal species or genus (*Malassezia* sp. unknown). **(B)** Relative abundance of all fungal genera, extended to the species level for the clinically important *Candida* species (colored dots), is plotted against the prevalence of each genus and *Candida* species in the six CF subject samples (pre- and post-treatment) and show that fungi that are highly abundant in a single patient are also highly prevalent across patients. The colored dots indicate the *Candida* species and the genus *Malassezia* that are both highly abundant and highly prevalent and the empty dots represent all remaining fungal genera identified in our study.

Datasets from each sputum sample contained an average of 21,434 fungal reads ranging from 1,672 fungal reads (the inter-treatment sample of subject #9) and 37,684 (the post-treatment sample of subject #6) (Additional file 3: Table S3), and a rarefaction analysis indicated that the sequencing depth was sufficient to detect the vast majority of fungi present (Additional file 4: Figure S2A). *Candida* represented the dominant genus with 11 different species. The top three *Candida* species (*C. albicans*, *Candida dubliniensis*, and *Candida parapsilosis*) accounted for 74%–99.9% of all ITS1 reads in all samples (Figure 1A) making these species both the most abundant species and also the most prevalent. *C. albicans* ranged from 0.1% of reads (the post-treatment sample from subject #2) to 98.8% of reads (the post-treatment sample from subject #6) (Additional file 5: Table S4). In three subjects, the only reported fungi using culture-based detection methods were *C. parapsilosis* in subjects #1 and #3 and *C. dubliniensis* in subject #2 (Additional file 1: Table S1). Another clinically interesting *Candida* species, *Candida tropicalis*, was detected at lower levels, ranging from 2 to 477 reads, and only in half of the samples (Figure 1B). Aside from *C. albicans*, only *Malassezia* spp. (including *Malassezia restricta* and *Malassezia globosa*) occurred in all samples, but at levels 10- to 50-fold lower than the *Candida* spp. reads (Figure 1A,B). We detected low numbers of reads corresponding to three different species of *Aspergillus* (*Aspergillus unguis*, *Aspergillus versicolor*, and *Aspergillus* sp.) in this study, but not *A. fumigatus*, another common CF pathogen, which we have previously detected in other CF sputum samples (data not shown). Across all 14 samples, our VAMPS-based analyses detected 111 different taxa at the genus level, and these resolved to 147 species level assignments (Additional file 5: Table S4). The minor members of the mycobiome are discussed in more detail below.

To validate the results obtained by GAST analyses, we employed BLAST to assign fungal ITS1 sequences to species and genus level categories (Additional file 6: Table S5). For the BLAST searches, we batch blasted examples of all unique reads and assigned a genus or species to each set of sequences. Comparison of GAST and BLAST results initially highlighted the need for database improvement (described above), and once the database had been modified, the two methods yielded a similar number of taxa. Because GAST reports the consensus annotation for all of the sequences that match the query, the developmental state of the ITS1 database may limit the assignment of genus or species level information. In this case, sequences clustered by GAST in VAMPS can be easily retrieved for further analysis.

Because the characterization of fungi within host-associated microbial communities is at an early stage, we sought to determine if there were any ITS1 sequences that have not been previously described. In initial analyses, up to 13.3% of sequences in a sample could not be identified as fungi (BLAST scores of greater than e - 15 for the top hit). On average, samples had 3.6% of reads in this category. Further analysis querying Genbank’s Nucleotide collection (nr/nt) database revealed that most of these unassigned sequences corresponded to ITS1 sequences in the genomes of food-associated plants such as *Pisum sativum* (peas), *Allium cepa* (onions), *Allium sativum* (garlic), *Solanum tuberosum* (potato*)*, and *Glycine max* (soybean) suggesting contamination of the sputum sample from subjects’ diets. The primers used to amplify fungal ITS1 sequences (Additional file 7: Table S2) displayed high levels of similarity to comparable regions in plants. VAMPS allows one to limit analyses to specific taxonomic levels or to specific groups (for instance, fungi or Ascomycetes) [53], and this eliminated the problem of contaminating sequences. A very small number of unassignable sequences (13 out of 300,072 reads) were also identified, but they were most often single reads that were only found within single samples and were not characterized further.

### Comparison of intra- and inter-species ITS1 sequence differences in *Candida* spp

Some fungal genomes may contain up to 600 copies of the ribosomal RNA genes (rDNA) and intraspecific ITS variability can exceed 24.2% [59-62]. In contrast, *C. albicans* typically has 150–200 copies of the ITS1 locus, with one report estimating less than 0.2% ITS variability within a strain [62]. Since *Candida* represented the predominant fungal genus in our CF subjects, we used Illumina MiSeq sequencing to investigate levels of ITS1 sequence variation within and among clinical *C. albicans* isolates. When the ITS1 region was amplified from genomic DNA from pure cultures of *C. albicans* wild type SC5314 and seven clinical isolates, then analyzed using Illumina MiSeq, the predominant sequence (>99% of reads) for each strain was the same (100% identical across all isolates). The second most abundant sequence accounted for only ~0.02% of reads. Similarly, we observed no evidence for intra-strain variation in the ITS1 sequences from the analysis of genomic DNAs isolated from pure cultures of three other *Candida* species (*C. dubliniensis*, *C. parapsilosis*, and *C. kruseii*) (data not shown).

When we compared the most abundant *C. albicans* ITS1 sequence in the sputum-derived datasets to the dominant sequence from the clinical isolates, we found that they were 100% identical (data not shown). Alignments of the three most common ITS1 sequences that resolved to *C. albicans* from each sample, with the ITS1 sequences from the genome information for *C. albicans* strains SC5314 and WO-1, displayed 99% identity. The second most abundant *C. albicans* ITS1 454 sequence amplified from sputum DNA differed from the most abundant ITS1 sequence by one missing “T” at position 60, which most likely reflects a 454 homopolymer sequencing error. By way of comparison, the closely related *Candida* species *C. albicans* and *C. dubliniensis* have ITS1 sequences that have 94.6% identity.

### Description of the mycobiome and bacterial microbiome across serial sputum samples

**Figure 2:**
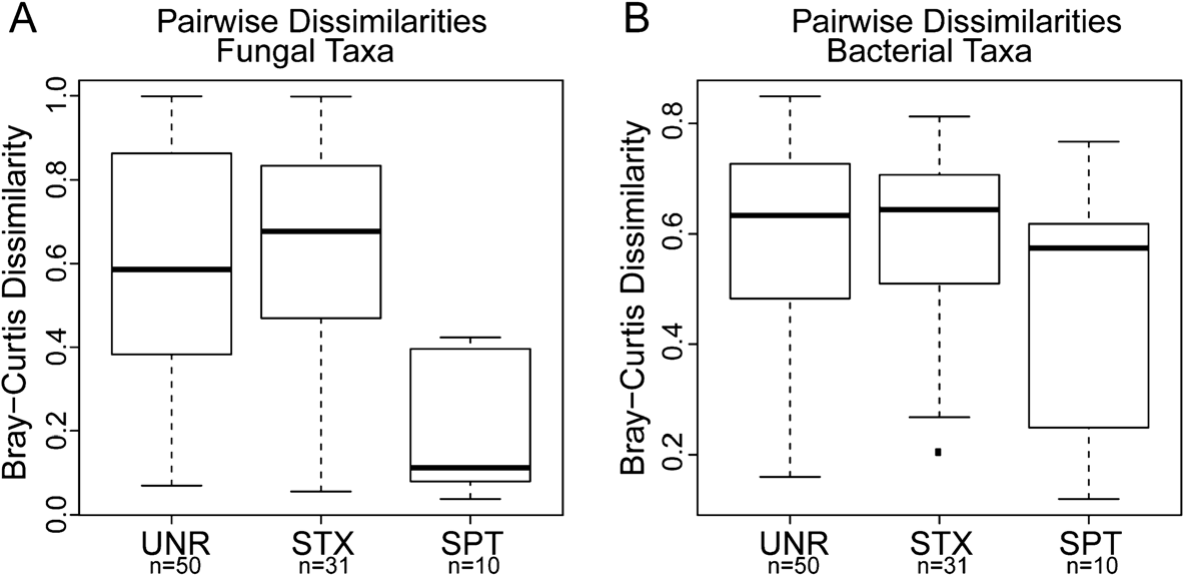
**Relatedness of microbial communities.** Box-and-whisker plots of pairwise Bray-Curtis distances of the mycobiome and microbiome of cystic fibrosis (CF) subjects. **(A)** Fungal samples from unrelated subjects on different treatments (UNR) are no more different than samples from unrelated subjects on the same treatments (STX). Mycobiome samples from the same subject (SPT) were significantly more similar to each other (*P* <0. 001) by a Tukey’s honest significant test than mycobiome samples from the same treatment group, suggesting that patients’ fungal communities are specific to patients and remain relatively stable during treatment. **(B)** Bacterial genera from the same subject (SPT) were marginally more similar than unrelated subjects on different treatments (UNR) or unrelated subjects on the same treatments (STX) but these differences were not significant by a Tukey’s honest significant test.

In the collection of serial samples, each subject provided a sputum sample at the time of admission for treatment of exacerbated respiratory disease using two or three antibacterials and one sample upon the completion of treatment; some subjects also provided a third sample during treatment. The drugs administered included Tobramycin, Vancomycin, Doripenem, Meropenem, Ceftazadime, and Linezolid and information which antibacterial drugs were used in each subject is provided in Additional file 1: Table S1. The fungal communities in all samples, grouped by subject, are shown in Figure 1A. The relative abundance of reads corresponding to the predominant *Candida* spp. appeared to be quite stable over time during treatment, and there was no obvious change upon treatment initiation (Figure 1A). Bray-Curtis dissimilarity and principal coordinate analysis were used to measure and represent taxonomic relatedness between classes of samples in the mycobiome and the microbiome samples. Antibiotic treatment did not establish a specific fungal community. Fungal samples from unrelated people at different points in therapy (unrelated, UNR) were no more different than samples from unrelated people at the same point in treatment (same treatment, STX). However, mycobiome samples from the same subject (same subject, SPT) were significantly more similar to each other (*P* <0.001) by a Tukey’s honest significant test (Figure 2A) than mycobiome samples from the same treatment point, suggesting that subjects’ fungal communities remained relatively stable. Furthermore, as shown in the PCA scores plot (Additional file 8: Figure S3), the samples from subjects #1, #6, and #8 clustered together as did those from subjects #2 and #9. Subject #3 was distinct from the other samples (Additional file 8: Figure S3).

**Figure 3:**
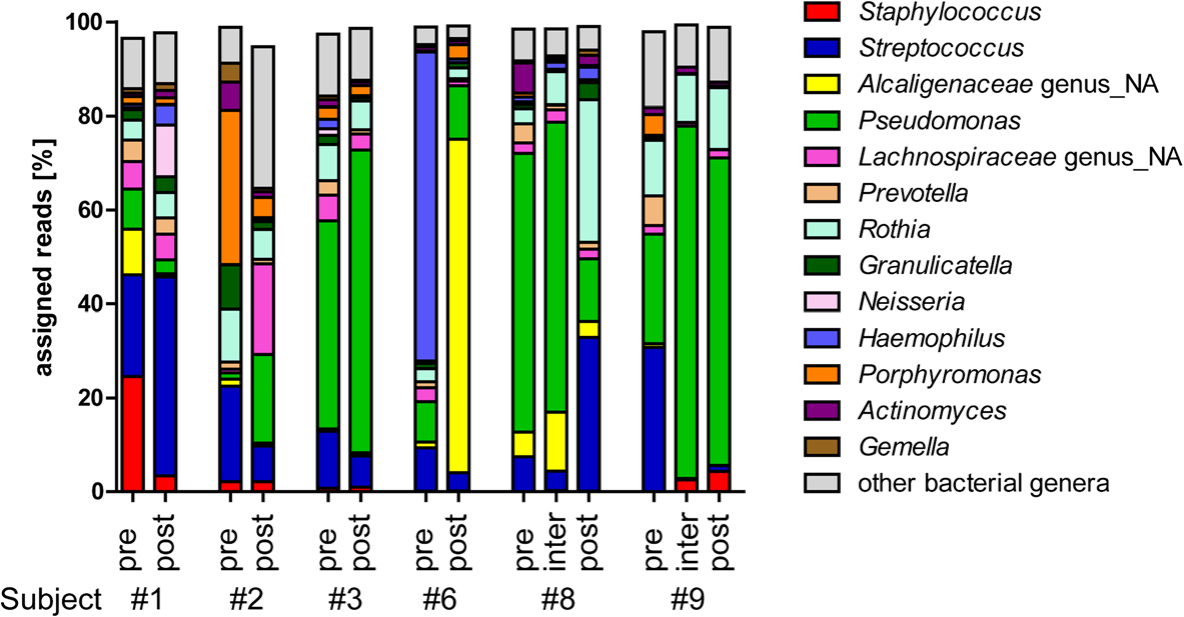
**Characterization of the bacterial communities of sputum samples from CF subjects.** Fraction of reads assigned to each of the top 13 bacterial genera detected in CF subjects before treatment (pre), during treatment (inter), or after completion of treatment (post).

To compare the fungal community profiles to those for the bacteria, we amplified and sequenced the V6 region of the bacterial 16S rDNA gene from the same sputum DNA samples. On average, we recovered 726,787 sequences from each sample (Additional file 3: Table S3), and a rarefaction analysis indicated that this deep sequencing effort identified most members of the sampled bacterial microbiomes (Additional file 4: Figure S2B). Ninety-nine percent of all reads from all samples were assigned to 44 genera, though not all 44 genera were detected in every sample (Additional file 9: Table S6). The remaining 1% of reads corresponded to 397 different genera. As reported by other studies, most samples contained high levels of the genera *Pseudomonas*, *Staphylococcus*, *Streptococcus*, *Prevotella*, *Rothia*, *Granulicatella*, *Neisseria*, *Haemophilus*, *Porphyromonas*, *Gemella*, and members of the families *Alcaligenaceae* and *Lachnospiraceae* (Figure 3) [2,4,63,64].

Consistent with published work [4,6,65], the bacterial community also displayed stability in the major community members in many subjects (Figure 3), though some changes in the bacteria with the highest relative abundance were observed in subjects #2 and #6 (Figure 3). The Bray-Curtis dissimilarity of the bacterial communities showed a less clear picture than for the fungi, but it still was apparent that the samples from the same subject showed some degree of relatedness. Bacterial community profiles from the same subject were marginally more similar (SPT) but the difference was not significant by a Tukey’s honest significant test (Figure 2B); like for the fungi, the STX samples were not more similar than the UNR samples. There was less of a clear trend in sample clustering in the PCA scores plot, and the larger number of bacterial genera in each sample may have contributed to this complexity (Additional file 8: Figure S3).

**Figure 4:**
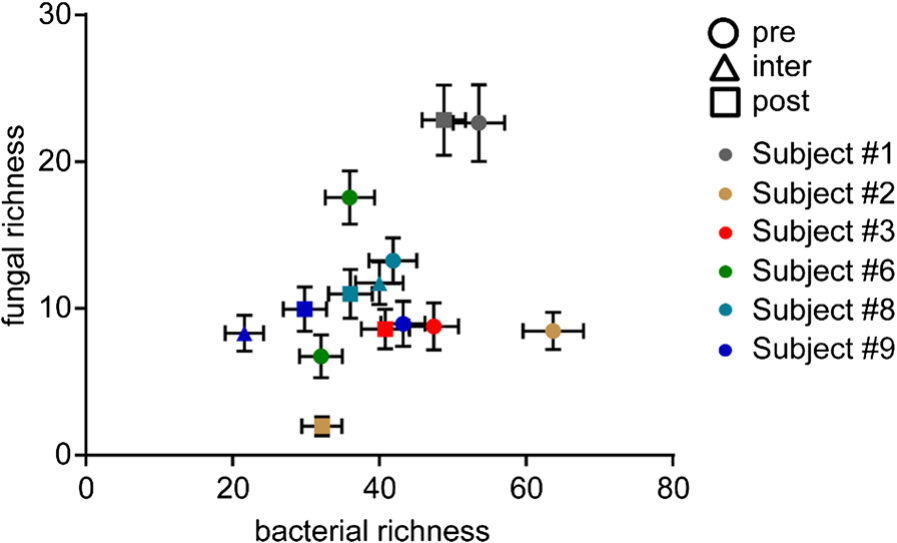
**Comparison of fungal and bacterial richness within samples.** Scatter plot of the subsampled mean normalized number of bacterial genera vs. the number of fungal genera found in the sputum of CF subjects. Neither treatment nor high numbers of bacterial or fungal genera have an obvious impact on each other. The pre-treatment samples are depicted as circles, the intermediate (inter) samples collected during treatment shown as triangles, and the post-treatment samples are depicted squares. The colors represent the different subjects.

To compare bacterial and fungal richness across samples, we first accounted for differences in the number of reads by subsampling 1,000 reads for each fungal and bacterial sample and repeatedly subsampling 1,000 times. First, we found that the normalized mean number of fungal genera did not correlate with the number of normalized mean bacterial genera (Figure 4). Using a Wilcoxon matched-pairs signed ranked test, it was found that the post-treatment samples had a lower mean bacterial genus richness (*P* <0.05) when compared to pre-treatment samples (Additional file 10: Figure S4). While the normalized mean richness for the fungi trended in the same direction, the differences were not statistically significant (*P* >0.1).

### Analysis of minor members of the mycobiome

The remaining fungal genera represented relatively low abundance populations that occurred in one or more samples (Figure 1B and Additional file 5: Table S4 for identities). When using the BLAST-based mycobiome analysis (see Additional file 11), the number of taxa identified within a sample ranged from 5 taxa (resolved to 3 species, 1 genus, and 1 cluster of unidentified sequences in the post-treatment sample from subject #2) to 60 taxa (resolved to 43 species, 16 genera, and 1 cluster of unidentified sequences in the pre-treatment sample of subject #1) (Additional file 12: Table S5). Minor members (<0.05% of annotated ITS1 sequences) of the mycobiome included other *Candida* species, *Trichosporon* (an emerging pathogen in CF subjects [27,66]), and the human pathogens *Rhodotorula*, *Fusarium*, and *Penicillium* (Additional file 5: Table S4).

**Figure 5:**
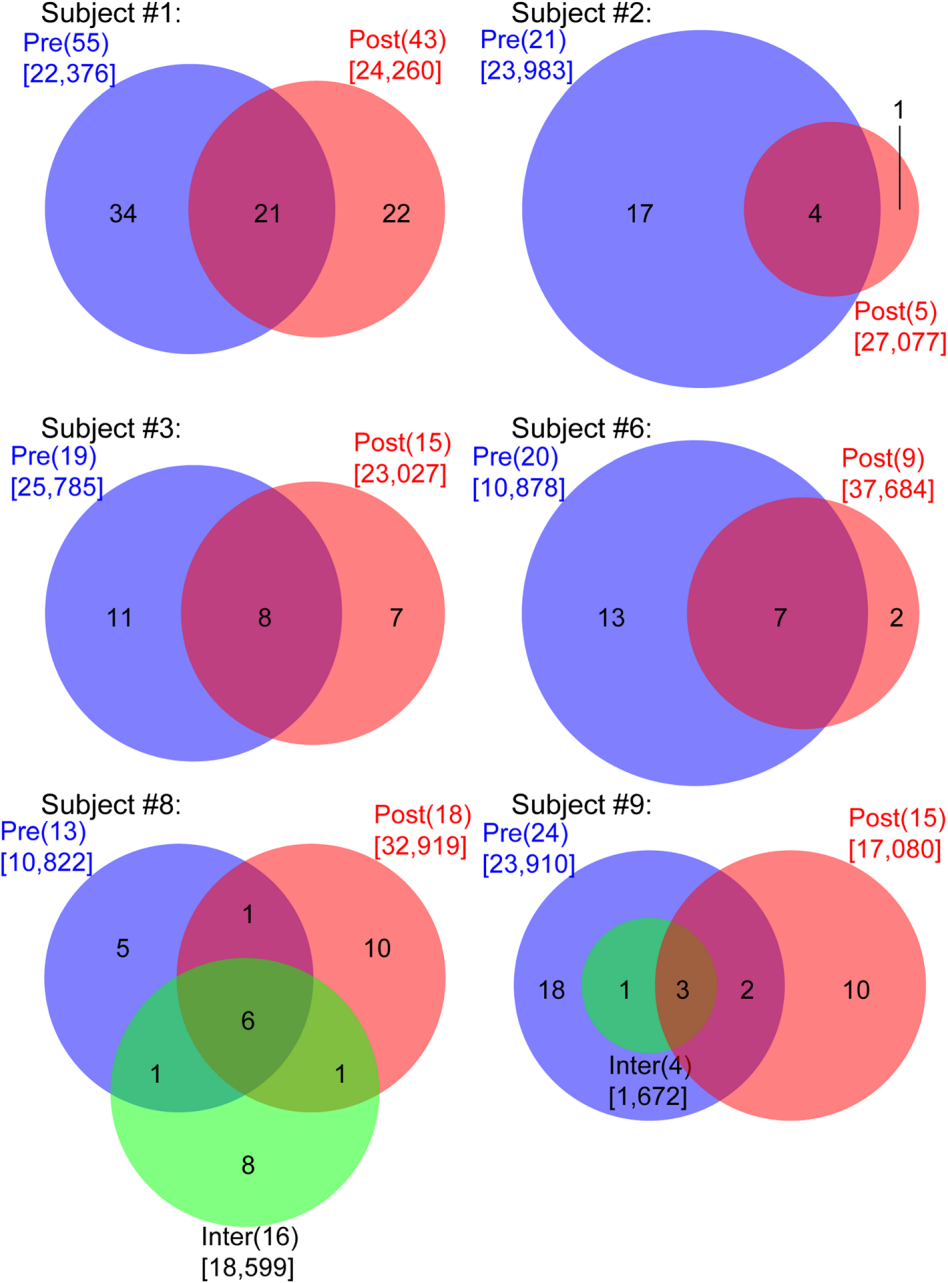
**Comparison of fungal communities in the same subject.** Distribution of fungal taxa detected in CF subjects either immediately after an exacerbation and before starting an antibacterial therapy (pre), approximately 2 weeks afterwards (post) or while hospitalized (inter) presented in Euler diagrams for pre-post samples (subjects #1–6) and Venn diagrams for pre-inter-post samples (subjects #8 and 9) diagrams. The numbers in parenthesis describe the total number of taxa detected in a sample; the numbers in the circles represent either the unique number of taxa in a sample or the number of shared taxa in the overlap regions. The numbers in brackets represent the total number of reads for each sample.

The deep sequencing in this study and the corresponding rarefaction analyses suggest that we have identified the number of taxa that were common to the two (and in some cases three) samples from a given subject (Additional file 4: Figure S2). The overlap in fungal taxa in samples from the same subject is presented as Euler and Venn diagrams (Figure 5 based on data in Additional file 5: Table S4). When only the pre- and post-treatment samples were compared, an average of 44% ±19% of the fungal species detected was common in both samples. This highlights the fact that some of the minor fungal taxa were not common across the serial samples and may represent transient species in the airway or oral community. As an example, 21 taxa were detected in both the pre-treatment and post-treatment samples from subject #1; 34 taxa were unique to the pre-treatment sample and 22 taxa were only in the post-treatment sample (Figure 5). As sequencing depth can impact the number taxa per sample and thus the apparent stability of a taxon, we also considered and presented the number of reads obtained for each sample; there were no differences in sequencing depth between the pre- and post-treatment samples from a given subject (Figure 5). The stability of species across samples from the same subject was also assessed for bacteria (Additional file 12: Figure S5). Again, comparing the pre- and post-treatment samples, we found that an average of 77% ±7% genera were in common in the two samples (Additional file 12: Figure S5 based on data in Additional file 9: Table S6). Additional file 13: Table S7 highlights those fungal taxa, including low abundance taxa that were detected in more than one sample.

### Analysis of the changes in community structure and absolute abundance before and after antibacterial therapy

**Figure 6:**
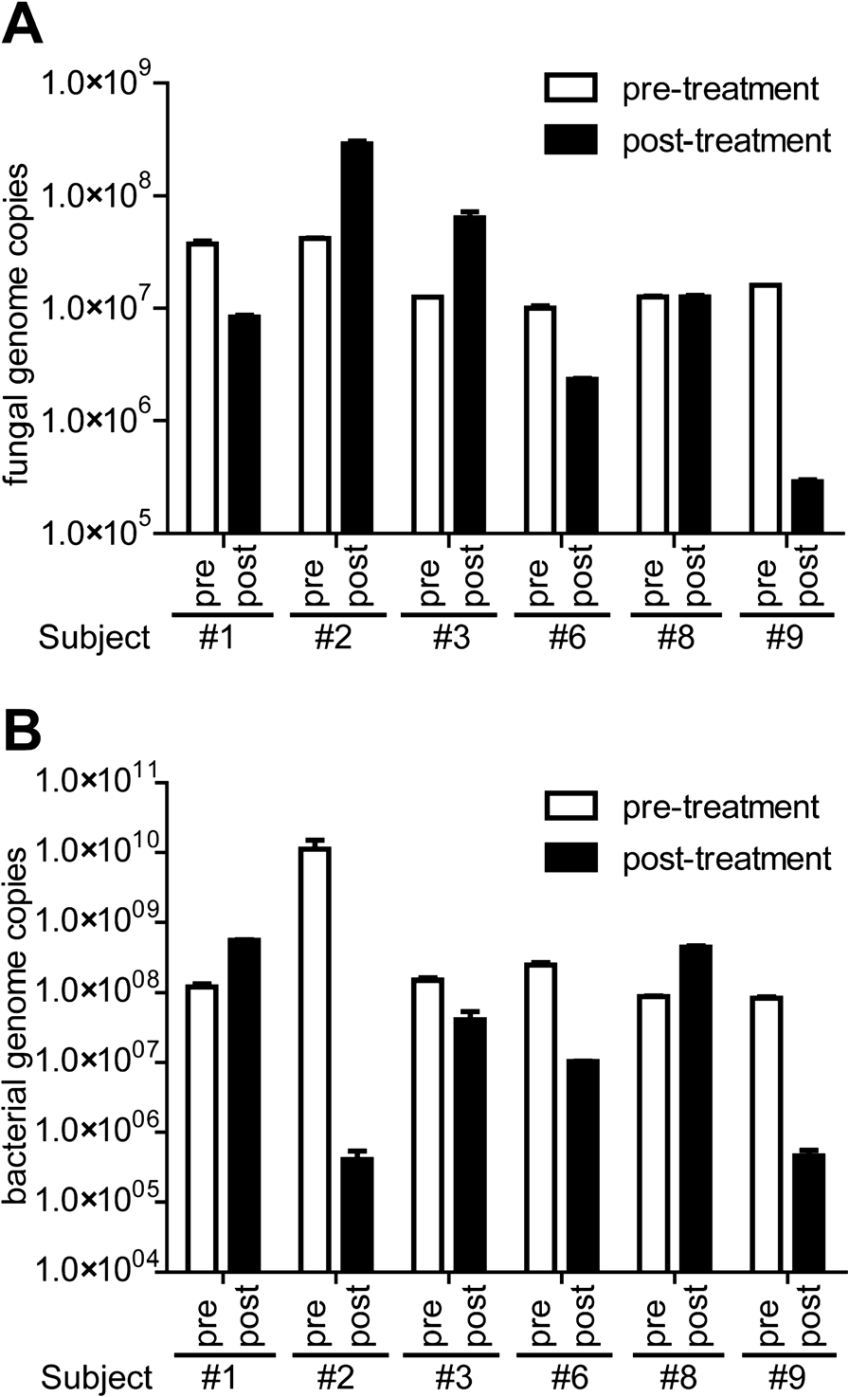
**Quantification of fungal and bacterial burden in CF subjects. (A)** Quantification of the fungal burden of sputum samples from CF subjects. Total fungal burden was determined by amplifying the 18S rDNA locus by qPCR. **(B)** Quantification of the bacterial burden of sputum samples from cystic fibrosis subjects. Total bacterial burden was determined by amplifying the 16 s rRNA locus by real-time qPCR.

To complement the data on the relative abundances pre- and post-treatment, we also analyzed the density of bacteria and fungi within each sputum sample by qPCR. Results were normalized to sputum dry weight (genome copies per mg dry weight) (Figure 6A). On average, we could detect ~4.2 × 10^7^ fungal genome copies/mg dry weight sputum with a maximum of 2.8 × 10^8^ copies in the post-treatment sample of subject #2 and minimum of 2.9 × 10^5^ copies in the post-treatment of subject #9 (Figure 6A). Overall, we did not observe a specific trend in fungal burden corresponding to the antibacterial therapy. In three subjects, the fungal burden dropped (subjects #1, #6, and #9); in two subjects, the fungal density increased (subjects #2 and #3); and subject #8 exhibited no difference. We designed primers to quantify levels of the four *Candida* species (Additional file 7: Table S2), and the primers were shown to be species specific with respect to one another in studies performed using genomic DNA from purified cultures (data not shown). When the absolute abundances of fungi, determined using 18S rDNA quantification was compared to the sum of the levels found using the *Candida* species specific primer sets, they were within the same order of magnitude (Additional file 14: Figure S6) confirming that the *Candida* species were numerically dominant among the fungi within these samples. The qPCR approach also confirmed that relative abundance of *C. tropicalis* levels was much lower than *C. albicans* (data not shown). This finding may parallel observations that *C. tropicalis* is often less abundant than the other *Candida* species in oral samples [67-70].

We determined total bacterial burden without pre-amplification by targeting the 16S rRNA locus [2] (Figure 6B). On average, we detected ~9.2 × 10^8^ bacterial genome copies/mg dry weight sputum with a maximum of 1.1 × 10^10^ copies in the pre-treatment sample of subject #2 and minimum of 4.0 × 10^5^ copies in the post-treatment of subject #2. As with the fungal quantification, we did not observe a specific trend in bacterial density in the pre- and post-treatment samples. The bacterial sputum density dropped in four of the patients, most significantly in subject #2 in which the bacterial burden within sputum dropped ~27,500-fold to a level close to the detection limit. In two subjects, the bacterial level went up approximately five-fold. There was no correlation between the changes in bacterial density and changes in fungal density (Figure 6).

## Discussion

Dominant members of fungal communities, which we defined as those that account for more than 80% of all assigned reads per sample (*Candida* and *Malassezia* species) (Figure 1A) persisted throughout treatment without significant change in relative abundance suggesting that there is stability within the mycobiome. Many minor members of the fungal community appeared to be ephemeral or sporadic, and thus did not show changes in prevalence or abundance that could be attributed to antibacterial treatment (Figure 5 and Additional file 13: Table S7). Based on the depth of sequencing and the rarefaction analyses, the apparent instability of minor members is unlikely an artifact of sequencing depth, but rather likely a reflection of their transient nature. The fungal species we detected within CF sputum mirrors results in other recently published work. For example, denaturing high-performance liquid chromatography analysis of the ITS1 region amplified from fungi within CF sputum also found *C. albicans*, *M. restricta* and *M. globosa*[71]. In a study of four stable CF subjects by Delhaes et al. [19], these fungi were also well represented. While *C. albicans* is commonly observed in CF sputum, the presence of *Malassezia* is less well understood in part because *Malassezia* cannot be cultured without the use of specialized medium. Investigation of the fungal communities on the human skin found *M. restricta* and *M. globosa* to be very common [72], and future work will be required to determine if *Malassezia* are living in the lung or if the signal is coming from dead cells or DNA. Mournier et al. [71] also detected more fungi using molecular methods when compared to culture-based analyses, and *Candida* and *Malassezia* were among them. In addition, they found that *A. fumigatus* was detected by culture in some samples but not by molecular techniques [71]. While *A. fumigatus* was not seen in clinical microbiological analyses of the sputa in this study, we were surprised by its complete absence from our datasets. We have detected *A. fumigatus* in other samples indicating that our methods can amplify its ITS1 region. A recent shotgun metagenomic analysis of CF sputum DNA also found significant levels of *C. albicans* and *A. fumigatus* by culture but found no DNA evidence for *A. fumigatus* and a small number of other *Candida* sequences [73]. In contrast to what was observed in CF sputum, Charlson et al. [74] analyzed the fungi in the lavages of healthy individuals, and they found that healthy volunteers’ bronchoalveolar lavage (BAL) yielded scant fungal amplification. Although *Candida* was identified in several healthy subjects’ oropharyngeal washes, it was absent in their BAL.

In this study, we explored whether inpatient treatment, which included multiple antibacterials, impacted the fungal community in a consistent way, perhaps indicating changes in biological niches or environmental conditions upon perturbation of the bacterial community. Our data suggest that the use of multiple antibiotics did not necessarily have strong effects on either the bacterial or fungal communities. While the number of bacterial genera (richness) declined slightly but significantly (Additional file 10: Figure S4), there was not a consistently change to the structure of the community with similar relative abundances of dominant species in four out of six subjects (Figure 2 and Additional file 12: Figure S5). In these same samples, we did not observe a consistent increase in the number of fungal genera (Additional file 10: Figure S4) and the fungal species that dominated the samples did not change markedly (Figure 1A). This lack of difference between pre- and post-samples may well be due to the fact that these subjects have been exposed to years of inhaled and intravenous antibacterial therapy, and the bacteria in their lungs have adapted or evolved in this context. Thus, future studies in subjects without routine exposure to antibiotics are needed in order to assess the effects of antibacterials on the resident fungi. We and others have characterized the interactions between *Pseudomonas aeruginosa* and *C. albicans*, including effects on fungal morphology [75,76], viability [77,78], metabolism [76,79], and virulence factor regulation [80,81], and the high relative abundance of *P. aeruginosa* in the bacterial communities and *C. albicans* in the fungal communities supports the hypothesis that these species have the potential to impact one another in the lung.

The therapy used in the treatment of disease exacerbation also had no consistent impact on fungal density in patient sputum samples. Measurements of fungal and bacterial genomes per milligram of sputum showed an inconsistent trend across serial samples collected before and after antibacterial treatment (Figure 6) suggesting the need for either a larger number of subjects or alternative methods for the measurement of live microbes including bacteria and fungi. Previous reports in other body sites have linked antibiotics to increased fungal burden [82-88]. In a recent study, Dolville et al. [89] investigated the gut microbiome of mice over 76 days of treatment with the antibiotics vancomycin, ampicillin, neomycin, and metronidazole and subsequent recovery. They observed that bacterial numbers dropped in abundance more than 3-fold while fungi increased ~40-fold in abundance upon treatment but found that both numbers went back to pre-treatment levels upon recovery. Interestingly, the bacterial microbiome was detectably different from *Candida*-free controls and *Candida* became more abundant than at the time point prior to treatment [89]. While we observed that fungal density in sputum had the potential to vary (either increase or decrease), we found no common trend. It is important to note that all of these patients have a significant history of prior antibiotic exposure and thus some adaptation may have occurred. The effects of antibiotic therapy in fungal populations may differ from those obtained upon first exposure to a drug.

Fungal load has been associated with lung disease severity [16,20,22] suggesting that it is important to measure fungal density in sputum in addition to the mere presence of specific species. We found a range of fungal loads across samples (Figure 6), and this range is consistent with previous reports. For example, Bauernfeind et al. [15] found between 10^4^ and 10^7^ colony forming units per gram of sputum of *Candida* using culture methods. Charlson et al. [74] highlight the importance of measuring bacterial and fungal loads and to compare it to background contamination by upper respiratory tract flora to identify lung-enriched taxa. Here, we applied qPCR quantification of the 16S rDNA locus for total bacteria, the 18S rDNA locus for total fungi, and species-specific primers to quantify three *Candida* species. One limitation of DNA-based quantification protocols is the inability to differentiate between nucleic acids from live and dead species. Methods to inactivate DNA from dead or lysed cells would enhance our understanding of the active microbiota in the lung [90].

Finally, we report the use of VAMPS for analysis of the ITS1 sequencing results. VAMPS, which has provided a user-friendly tool for the analysis of bacterial communities [2,91], now has an improved database that will expand the ability to analyze fungal ITS1 sequences from clinical samples. The VAMPS database, derived from the UNITE database [58], contains most of the available ITS1 sequences. However, further curation will improve taxonomic information in existing entries, and the inclusion of additional high-quality, taxonomically resolved sequences that further aid in the analysis and taxon assignment in future human mycobiome studies [53].

## Conclusions

The data presented in this report found stable fungal communities in the sputum of six individuals with CF. Though to a lesser extent, stability was also mirrored in the bacterial communities. We hypothesize that the resident bacteria have developed a tolerance or resistance to antimicrobials due to repeated prior exposure, and thus, little perturbation of co-colonizing fungi is observed. In addition to numerically dominant fungal populations that are predominantly members of the genus *Candida*, there are less abundant fungi, many of which were less stable across serial samples from the same subject. To enhance future analyses of the mycobiome, we developed and modified a fungal ITS1 database for use in VAMPS, a publically available tool for the analyses of visualization of data on microbial communities. Future studies will continue to investigate the effects of antibacterial drugs on fungal and bacterial organisms and their densities in sputum.

## Methods

### Study participants and ethics statement

This study was performed in accordance with the guidelines set by the Committee for the Protection of Human Subjects (CPHS) and approved by the Institutional Review Board (IRB) at Dartmouth College. Sputum samples were obtained from six subjects over the age of 18 with a confirmed diagnosis of CF and who had previous cultures positive for *P. aeruginosa* after written, informed consent. Information about the study participants is provided in Additional file 1: Table S1.

### Sample collection

Serial sputum samples were collected from six CF subjects at the time of hospitalization for treatment of a respiratory disease exacerbation and after a complete course of intravenous antibacterial therapy, which often included the use of multiple antibiotics (Additional file 1: Table S1). For some subjects, an intermediate sample during therapy was also collected. Study participants expectorated sputum into a sterile specimen cup, and the sputum sample was stored at -80°C. In preparation for DNA extraction, the sample was thawed and homogenized by passing the sputum multiple times through needles of increasing gauges. Sputum (500 μl) was dispensed into a 2-ml screw cap tube and frozen at -80°C for at least 1 h prior to lyophilization for at least 12 h. The dry weight of the sputum was determined before DNA extraction.

### DNA extraction

Cells within the dried sputum sample were disrupted using a mixture sterile glass beads (equivalent amounts of 0.1-, 0.5-, and 1-mm diameter beads) in a bead beater (Biospec, Bartlesville, OK, USA); the tubes were agitated four times for 30 s each time with a cooling step in between pulses. The ground samples were resuspended in 300 μl of TE + DTT (TE amended with DTT at a final concentration of 0.08% added from a 2% stock solution) containing lysozyme (3 mg/ml) and lyticase (10 U/ml) and incubated for 30 min at 37°C. Cell lysis buffer (500 μl) (Qiagen Puregene Core Kit B, QIAGEN Inc., Valencia, CA, USA) was added, and the mixture was incubated for 15 min at 80°C. To remove RNA, RNase (1.5 μl) (QIAGEN Inc.) was added and the samples were incubated for 30 min at 37°C. Lysates were chilled on ice for 1 min, 200 μl of Protein Precipitation Solution (Qiagen Puregene Core Kit B, QIAGEN Inc.) was added, and the solutions were mixed vigorously for 20 s. Cell debris was sedimented by centrifugation at 13,000 rpm for 3 min, and the supernatant was transferred to a new 1.5-ml tube prior to addition of 600 μl of 100% isopropanol. After mixing by inversion, the DNA was precipitated by centrifugation at 13,000 rpm for 20 min. The DNA pellet was washed with 300 μl of 70% ethanol and air dried before resuspension in 100–200 μl of DNA hydration solution (Qiagen Puregene Core Kit B, QIAGEN Inc.). The DNA concentrations were measured using a Nanodrop.

### Fungal ITS1 amplification and sequencing

The ITS1 region was amplified from sputum template DNA using the primers ITS1F and ITS1R (Additional file 7: Table S2) in two rounds of PCR. The reaction components were 1× Qiagen PCR buffer, 0.75 mM MgCl_2_, 7% (*v*/*v*) dimethyl sulfoxide (DMSO), 0.2 mM deoxynucleotide triphosphates (dNTPs), 0.4 μM primers, 1.25 units Taq polymerase (QIAGEN Inc.), 100 ng of template DNA, and water in a 25-μl reaction volume. Cycling conditions were 95°C 5 min; 95°C 30 s, 50°C 30 s, 72°C 45 s for 10 cycles; and 72°C 2 min. PCR products were purified using the MinElute PCR Purification Kit (QIAGEN Inc.) and resuspended in 10 μl of EB buffer. The entire 10 μl volume was used as template for a second round of PCR using primers containing adapter and barcode sequences for Roche GS-FLX amplicon pyrosequencing (Additional file 2: Table S2). Reaction components and cycling conditions were as described above, but with 30 cycles. Amplified DNA was purified with 0.7 volumes of Agencourt AMPure XP beads (Beckman Coulter, Brea, CA, USA) and quantified on a Bioanalyzer 2100 (Agilent, Palo Alto, CA, USA). Purified amplicon libraries were sequenced using the Titanium amplicon protocol on a GS-FLX (Roche Applied Science, Indianapolis, IN, USA) following the methods outlined in [2]. Sequence data were quality filtered as described in [54,92]. We identified and removed chimeric reads by querying against the curated reference ITS1 database, as well as using the sequences as seeds in UCHIME *de novo*[93]. The raw sequencing results of the ITS1 analysis are deposited in the Sequence Read Archive (NCBI BioProject PRJNA246028; National Center for Biotechnology Information (NCBI), US National Library of Medicine, Bethesda, MD, USA) and are summarized in Additional file 5: Table S4.

For the MiSeq analysis of the ITS1 region amplified from clinical isolates of *Candida* spp., template DNA was isolated using the Epicentre Masterpure Yeast DNA Purification Kit. Primers are listed in Additional file 7: Table S2. The amplification and sequencing methods used are comparable to those reported [94].

### Bacterial 16S v6 amplification and sequencing

The bacterial communities from the pre- and post-antibacterial treated CF subjects were characterized by Illumina HiSeq 16S rRNA V6 amplicon sequencing (Illumina Inc., San Diego, CA, USA) as previously described by Eren et al. [95]. Briefly, we amplified the bacterial V6 region from each sample in triplicate 33 μL reactions, pooled the triplicates, and verified successful amplification on a Caliper LabChip GX (Perkin Elmer, Waltham, MA, USA). We cleaned amplicon products with a QIAQuick clean up assay (QIAGEN Inc.), and used fusion V6 primers to barcode each sample in a multiplexing strategy. Final barcoded products were quantitated with a Picogreen assay (Invitrogen/Life Technologies, Grand Island, NY, USA) and pooled in equimolar concentrations with respect to the target size. All PCR reactions contained 1× HiFi Buffer, 2 mM MgSO_4_, 0.02 U/μL Platinum Taq polymerase (Invitrogen), 0.2 mM each dNTPs (ThermoFisher Scientific, Milwaukee, WI, USA), and up to 5 ng template. The final pool was size selected (Pippin Prep, SageScience, Beverly, MA, USA), quantitated (Kapa Biosystems, Woburn, MA, USA) and sequenced on a HiSeq 1000 in a 2 × 100 nt sequencing run. Quality filtering and chimera checks were performed as described above. The raw sequencing results of the bacterial 16S v6 analysis are deposited in the Sequence Read Archive (NCBI BioProject PRJNA256169; National Center for Biotechnology Information (NCBI), US National Library of Medicine, Bethesda, MD, USA) and are summarized in Additional file 9: Table S6.

### Microbiome and mycobiome clustering and taxonomic assignment

The website http://vamps.mbl.edu provides access to individual reads, taxon assignments, and descriptions of individual clusters. To create the databases that are accessed through VAMPS researchers at the Marine Biology Laboratories at Woods Hole merged paired-end 100 bp reads into single consensus reads that completely covered the V6 region, and they assigned taxonomy with GAST [54] against a curated SILVA database [96]. To compare operational taxonomic unit (OTU) clustering performance, they employed the default UCLUST method [97] at a 97% similarity threshold with minimum cluster size of 2 using quantitative insights into microbial ecology (QIIME) (v1.5) [98].

### Rarefaction analysis

To verify adequate sequencing depth of the mycobiome and the microbiome, we performed rarefaction analysis of the two datasets. For both rarefaction analyses, we used raw counts of all subject samples generated by VAMPS with the taxonomy depth selector set to “species” to create a read matrix. This table was rarefied using an R-based protocol described on the webpage “Individual Based Rarefaction using R-package” (http://www.jennajacobs.org/R/rarefaction.html) [99]. The resulting table was transferred into GraphPad Prism 6 to plot the rarefaction curves.

### Euler and Venn diagrams

To visualize the impact of antibacterial treatment on the prevalence of fungal and bacterial taxa detected in CF subjects either immediately after an exacerbation and before starting an antibacterial therapy (pre), approximately 2 weeks afterwards (post) or while hospitalized (inter), we used the Additional file 5: Tables S4 and Additional file 9: Table S6 to generate the diagrams. First, we copied the data for pre, post and if applicable inter samples into the list-fields of an online Venn diagram generator (http://www.bioinformatics.lu/venn.php). Using the R package “RVennDiagram” (http://cran.r-project.org/web/packages/VennDiagram/index.html), we generated Euler (“draw.pairwise.venn”) and Venn (“draw.triple.venn”) diagrams following the instructions in the manual (http://cran.r-project.org/web/packages/VennDiagram/VennDiagram.pdf).

### Quantification of fungi, *Candida* species, and bacteria

In order to quantify minor members of the mycobiome, we performed analyses using either sputum DNA or pre-amplified DNA derived from ten rounds of PCR starting with 100 ng of sputum DNA using primers specific to a conserved region of the 18S rDNA (FungiQuant primers) [100] or *Candida* species-specific primers (Additional file 7: Table S2). We confirmed that the pre-amplification step was linear with respect to the initial sample; we performed multiple control experiments using genomic DNA isolated from different fungal species at concentrations ranging from 30 pg/μl to 300 ng/μl. The preamplification allowed for the analysis of minor members of the mycobiota. The reaction components of the first PCR were 1× Qiagen PCR buffer, 0.75 mM MgCl_2_, 7% (*v*/*v*) DMSO, 0.2 mM dNTPs, 0.4 μM primers, 1.25 units Taq polymerase (QIAGEN Inc.), 100 ng sputum DNA, and water in a 25 μl reaction volume. Cycling conditions were 95°C 5 min; 95°C 30 s, 50°C 30 s, 72°C 45 sec for 10 cycles; and 72°C 2 min. These PCR products were purified using the MinElute PCR Purification Kit (QIAGEN Inc.), and resuspended in 10 μl of EB buffer. Quantitative PCR (qPCR) was conducted in 20 μl reaction volumes with the iQ SYBR Green Supermix (Bio-Rad Laboratories, Hercules, CA, USA). Reactions contained 2 μl of the amplicon suspension from the first round PCR and 0.2 μM of the appropriate primers. To quantify total fungal burden, we used the primers FungiQuant_RT_F and FungiQuant_RT_R [99]. The aforementioned species-specific qPCR primers were designed to assess *Candida* burdens based on gene sequences available from the *Candida* Genome Database (http://www.candidagenome.org/). Genes used for primer design were CAWG_05066 (*C. albicans*), CPAR2_301290 (*C. parapsilosis*), Cd36_16280 (*C. dubliniensis*) and CTRG_03824 (*C. tropicalis*) and were selected based on conservation within species and a lack of cross reactivity with bacterial, fungal, and human sequences. qPCR was performed utilizing a CFX96 real-time PCR detection system combined with a C1000 thermal cycler (Bio-Rad Laboratories). All PCRs were done in duplicate, and data were analyzed with the CFX96 System gene expression software. Melt curve analysis was performed after the PCR was complete to confirm the absence of nonspecific amplification products. Standard curves containing a known numbers of genome equivalents were used to calculate genome numbers per mg dry weight.

For total bacterial quantification, 100 ng of sputum DNA was used as the PCR reaction template. To generate a standard curve for quantification purposes we prepared 10-fold dilutions of *P. aeruginosa* genomic DNA isolated from 10^2^ to 10^6^ cells*.* The 16S rDNA genes were amplified by qPCR, using the universal bacterial primers “total bacteria_F” and “total bacteria_R” at a final concentration of 0.2 μM [101,102] (Additional file 7: Table S2). qPCR was conducted in 20 μl reaction volumes with the iQ SYBR Green Supermix (Bio-Rad Laboratories). qPCRs for each sputum sample were performed and analyzed on the CFX96 PCR system as described above.

### Statistical analysis

Bray-Curtis dissimilarity was measured using the ecodist [103] and vegan [104] packages in R statistical software. Significant differences between groups were established using analysis of variance with Tukey’s honest significant difference post-test. Tests producing a *P* value less than 0.05 were deemed significant. The program Prism 6 (GraphPad, San Diego, CA, USA) was used for remaining statistical tests.

## Abbreviations

CF: cystic fibrosis; GAST: Global Alignment for Sequence Taxonomy; ITS1: internal transcribed spacer 1; MBL: Marine Biology Laboratories at Woods Hole; OTU: operational taxonomic unit; PCR: polymerase chain reaction; QIIME: quantitative insights into microbial ecology; qPCR: quantitative PCR; rDNA: ribosomal RNA genes; VAMPS: visualization and analysis of microbial population structures.

## Competing interests

The authors declare that they have no competing interests.

## Authors’ contributions

AA, LAM, and EFD collected sputum samples. SDW, with important contributions from EFD, prepared sputum samples for deep sequencing analysis. SDW performed the qPCR analysis of fungi DNA, and SDW and LMF in consultation with GAO performed the qPCR analyses of bacterial DNA. THH performed the statistical analyses. SDW, HGM, SLG, and MLS performed the deep sequencing and its analysis. SDW, AS, and MLS modified and refined the fungal database for usage in VAMPS. DAH and AA contributed to study design. SDW and DAH wrote the manuscript with important contributions from coauthors, especially HLM and MLS. All authors read and gave approval to the final manuscript.

## Supplementary Material

Additional file 1: Table S1 Microbiological results from samples collected at the time of admission for treatment of exacerbation and antibacterial drugs administered. Click here for file

Additional file 2: Figure S1 Analysis of amplification and sequencing method reproducibility. DNA from a sputum sample was divided, amplified, sequenced, and analyzed in parallel to evaluate our methods. Click here for file

Additional file 3: Table S3 Total number of reads used in the mycobiome (fungi) and the bacterial microbiome analyses. Click here for file

Additional file 4: Figure S2 Rarefaction analysis of the mycobiome and microbiome. Rarefaction curves to determine the completeness of deep sequencing of the mycobiome (A) and the microbiome (B). Repeated samples of species subsets were used to evaluate whether further sampling would likely yield additional species, as indicated by whether the curve has not yet reached a plateau. The *y*-axis indicates the number of species detected and the *x*-axis the number of sequences analyzed per sample. Click here for file

Additional file 5: Table S4 Fungal sequences detected in CF sputum samples using assignments made through VAMPS. The data are presented as the percent of total reads and absolute read number. Click here for file

Additional file 6: Table S5 Fungal species within CF sputum samples using the local BLAST analysis method. The data are normalized to percent of total reads. Click here for file

Additional file 7: Table S2 Primer list. Click here for file

Additional file 8: Figure S3 Principal component analysis based on Bray-Curtis dissimilarity. Bray-Curtis dissimilarity and principal coordinate analysis were used to measure and represent taxonomic relatedness between classes of samples in the mycobiome (fungal taxa) and the microbiome (bacterial taxa) samples. Pre-treatment samples are colored in red and are labeled A, intermediate samples collected during treatment are colored blue and are labeled B, and post-treatment samples are colored green and are labeled C. The numbers indicate the subject number. Click here for file

Additional file 9: Table S6 Bacterial genera identified in CF sputum samples using VAMPS. The data are presented as the percent of total reads and absolute read number. Only the genera that account for 99% of all reads are presented. Click here for file

Additional file 10: Figure S4 Comparison of the normalized richness of the mycobiome and microbiome. Comparison of the normalized mean fungal and bacterial richness of pre-treatment and post-treatment samples. The normalized mean fungal richness shows no significant difference between treatments (*P* >0.1) but the normalized mean bacterial richness decreases upon antibacterial treatment (*P* <0.05) (Wilcoxon matched-pairs signed rank test). Click here for file

Additional file 11 Mycobiome data analysis. Click here for file

Additional file 12: Figure S5 Bacterial communities within a subject before, during, and after treatment. Distribution of bacterial taxa detected in CF subjects either immediately after an exacerbation and before starting an antibacterial therapy (pre), approximately 2 weeks afterwards (post) or while hospitalized (inter) presented in Euler diagrams for the pre- and post-samples (subjects #1–6) and Venn diagrams for series with three samples (subjects #8 and 9). The numbers in parenthesis describe the total number of taxa detected in a sample; the numbers in the circles represent either the unique number of taxa in a sample or the number of shared taxa in the overlap regions. Click here for file

Additional file 13: Table S7 Summary of fungal taxa found in more than one sample. Click here for file

Additional file 14: Figure S6 Quantification of total fungi and specific *Candida* species in sputum of CF subjects. Quantification of the fungal burden of sputum samples from CF subjects. Fungal burden was determined by amplifying the 18S rDNA locus by qPCR. For *Candida* species-specific quantification, we used the genes CAWG_05066 (*C. albicans*), CPAR2_301290 (*C. parapsilosis*), and Cd36_16280 (*C. dubliniensis*). Fungal genome copy number was determined by comparison to a standard curve generated using DNA isolated from pure cultures of *Candida albicans* as described in the methods. Click here for file

## References

[B1] Tunney MM Klem ER Fodor AA Gilpin DF Moriarty TF McGrath SJ Muhlebach MS Boucher RC Cardwell C Doering G Elborn JS Wolfgang MC Use of culture and molecular analysis to determine the effect of antibiotic treatment on microbial community diversity and abundance during exacerbation in patients with cystic fibrosis Thorax 2011 66 7 579 584 10.1136/thx.2010.137281 21270069 PMC12747715

[B2] Filkins LM Hampton TH Gifford AH Gross MJ Hogan DA Sogin ML Morrison HG Paster BJ O’Toole GA Prevalence of streptococci and increased polymicrobial diversity associated with cystic fibrosis patient stability J Bacteriol 2012 194 17 4709 4717 10.1128/JB.00566-12 22753064 PMC3415522

[B3] Spilker T Vandamme P Lipuma JJ A multilocus sequence typing scheme implies population structure and reveals several putative novel *Achromobacter* species J Clin Microbiol 2012 50 9 3010 3015 10.1128/JCM.00814-12 22785192 PMC3421806

[B4] Zhao J Schloss PD Kalikin LM Carmody LA Foster BK Petrosino JF Cavalcoli JD VanDevanter DR Murray S Li JZ Young VB LiPuma JJ Decade-long bacterial community dynamics in cystic fibrosis airways Proc Natl Acad Sci U S A 2012 109 15 5809 5814 10.1073/pnas.1120577109 22451929 PMC3326496

[B5] Sibley CD Parkins MD Rabin HR Duan K Norgaard JC Surette MG A polymicrobial perspective of pulmonary infections exposes an enigmatic pathogen in cystic fibrosis patients Proc Natl Acad Sci U S A 2008 105 39 15070 15075 10.1073/pnas.0804326105 18812504 PMC2567494

[B6] Fodor AA Klem ER Gilpin DF Elborn JS Boucher RC Tunney MM Wolfgang MC The adult cystic fibrosis airway microbiota is stable over time and infection type, and highly resilient to antibiotic treatment of exacerbations PLoS One 2012 7 9 e45001 10.1371/journal.pone.0045001 23049765 PMC3458854

[B7] Stressmann FA Rogers GB van der Gast CJ Marsh P Vermeer LS Carroll MP Hoffman L Daniels TW Patel N Forbes B Bruce KD Long-term cultivation-independent microbial diversity analysis demonstrates that bacterial communities infecting the adult cystic fibrosis lung show stability and resilience Thorax 2012 67 10 867 873 10.1136/thoraxjnl-2011-200932 22707521

[B8] Leclair LW Hogan DA Mixed bacterial-fungal infections in the CF respiratory tract Med Mycol 2010 48 O1 S125 S132 10.3109/13693786.2010.521522 21067324

[B9] Morales DK Hogan DA *Candida albicans* interactions with bacteria in the context of human health and disease PLoS Pathog 2010 6 4 e1000886 10.1371/journal.ppat.1000886 20442787 PMC2861711

[B10] Peleg AY Hogan DA Mylonakis E Medically important bacterial-fungal interactions Nat Rev Microbiol 2010 8 5 340 349 10.1038/nrmicro2313 20348933

[B11] Hoppe JE Theurer-Mainka U Stern M Comparison of three methods for culturing throat swabs from cystic fibrosis patients J Clin Microbiol 1995 33 7 1896 1898 7665666 10.1128/jcm.33.7.1896-1898.1995 PMC228293

[B12] Bakare N Rickerts V Bargon J Just-Nubling G Prevalence of *Aspergillus fumigatus* and other fungal species in the sputum of adult patients with cystic fibrosis Mycoses 2003 46 1–2 19 23 12588478 10.1046/j.1439-0507.2003.00830.x

[B13] Haase G Skopnik H Groten T Kusenbach G Posselt HG Long-term fungal cultures from sputum of patients with cystic fibrosis Mycoses 1991 34 9–10 373 376 1820515 10.1111/j.1439-0507.1991.tb00797.x

[B14] Hughes WT Kim HK Mycoflora in cystic fibrosis: some ecologic aspects of *Pseudomonas aeruginosa* and *Candida albicans* Mycopathol Mycol Appl 1973 50 3 261 269 10.1007/BF02053377 4199669

[B15] Bauernfeind A Bertele RM Harms K Horl G Jungwirth R Petermuller C Przyklenk B Weisslein-Pfister C Qualitative and quantitative microbiological analysis of sputa of 102 patients with cystic fibrosis Infection 1987 15 4 270 277 10.1007/BF01644137 3117700

[B16] Chotirmall SH O’Donoghue E Bennett K Gunaratnam C O’Neill SJ McElvaney NG Sputum *Candida albicans* presages FEV1 decline and hospitalized exacerbations in cystic fibrosis Chest 2010 138 5 1186 1195 10.1378/chest.09-2996 20472859

[B17] Doern GV Brogden-Torres B Optimum use of selective plated media in primary processing of respiratory tract specimens from patients with cystic fibrosis J Clin Microbiol 1992 30 10 2740 2742 1400978 10.1128/jcm.30.10.2740-2742.1992 PMC270512

[B18] Güngör O Tamay Z Güler N Erturan Z Frequency of fungi in respiratory samples from Turkish cystic fibrosis patients Mycoses 2012 56 2 123 129 22747891 10.1111/j.1439-0507.2012.02221.x

[B19] Delhaes L Monchy S Frealle E Hubans C Salleron J Leroy S Prevotat A Wallet F Wallaert B Dei-Cas E Sime-Ngando T Chabe M Viscogliosi E The airway microbiota in cystic fibrosis: a complex fungal and bacterial community–implications for therapeutic management PLoS One 2012 7 4 e36313 10.1371/journal.pone.0036313 22558432 PMC3338676

[B20] Navarro J Rainisio M Harms HK Hodson ME Koch C Mastella G Strandvik B McKenzie SG Factors associated with poor pulmonary function: cross-sectional analysis of data from the ERCF. European Epidemiologic Registry of Cystic Fibrosis Eur Respir J 2001 18 2 298 305 10.1183/09031936.01.00068901 11529288

[B21] Storey DG Ujack EE Rabin HR Mitchell I *Pseudomonas aeruginosa lasR* transcription correlates with the transcription of *lasA*, *lasB*, and *toxA* in chronic lung infections associated with cystic fibrosis Infect Immun 1998 66 6 2521 2528 9596711 10.1128/iai.66.6.2521-2528.1998 PMC108233

[B22] Amin R Dupuis A Aaron SD Ratjen F The effect of chronic infection with *Aspergillus fumigatus* on lung function and hospitalization in patients with cystic fibrosis Chest 2010 137 1 171 176 10.1378/chest.09-1103 19567494

[B23] Masoud-Landgraf L Badura A Eber E Feierl G Marth E Buzina W Modified culture method detects a high diversity of fungal species in cystic fibrosis patients Med Mycol 2013 52 2 179 186 23651180 10.3109/13693786.2013.792438

[B24] Parize P Billaud S Bienvenu AL Bourdy S le Pogam MA Reix P Picot S Robert R Lortholary O Bouchara JP Durieu I Impact of *Scedosporium apiospermum* complex seroprevalence in patients with cystic fibrosis J Cyst Fibros 2014 doi:10.1016/j.jcf.2014.01.011 10.1016/j.jcf.2014.01.011 24530191

[B25] Bernhardt A Sedlacek L Wagner S Schwarz C Wurstl B Tintelnot K Multilocus sequence typing of *Scedosporium apiospermum* and *Pseudallescheria boydii* isolates from cystic fibrosis patients J Cyst Fibros 2013 12 6 592 598 10.1016/j.jcf.2013.05.007 23764085

[B26] Packeu A Lebecque P Rodriguez-Villalobos H Boeras A Hendrickx M Bouchara JP Symoens F Molecular typing and antifungal susceptibility of *Exophiala* isolates from patients with cystic fibrosis J Med Microbiol 2012 61 Pt 9 1226 1233 22580912 10.1099/jmm.0.042317-0

[B27] Hickey PW Sutton DA Fothergill AW Rinaldi MG Wickes BL Schmidt HJ Walsh TJ *Trichosporon mycotoxinivorans*, a novel respiratory pathogen in patients with cystic fibrosis J Clin Microbiol 2009 47 10 3091 3097 10.1128/JCM.00460-09 19656976 PMC2756937

[B28] Nagano Y Elborn JS Millar BC Walker JM Goldsmith CE Rendall J Moore JE Comparison of techniques to examine the diversity of fungi in adult patients with cystic fibrosis Med Mycol 2010 48 1 166 76 e1 10.3109/13693780903127506 19672783

[B29] Garcia L Respiratory cultures from Cystic Fibrosis patients, in Clinical Microbiology Procedures Handbook 2010 ASM Press

[B30] Price KE Hampton TH Gifford AH Dolben EL Hogan DA Morrison HG Sogin ML O’Toole GA Unique microbial communities persist in individual cystic fibrosis patients throughout a clinical exacerbation Microbiome 2013 1 1 27 10.1186/2049-2618-1-27 24451123 PMC3971630

[B31] Jubin V Ranque S Stremler N Le B Sarles J Dubus JC Risk factors for *Aspergillus* colonization and allergic bronchopulmonary aspergillosis in children with cystic fibrosis Pediatr Pulmonol 2010 45 8 764 771 10.1002/ppul.21240 20597074

[B32] Burns J Van Dalfsen J Shawar R Otto K Garber R Quan J Montgomery A Albers G Ramsey B Smith A Effect of intermittent administration of inhaled tobramycin on respiratory microbial flora in patients with cystic fibrosis J Infect Dis 1999 179 1190 1196 10.1086/314727 10191222

[B33] Cheer SM Waugh J Noble S Inhaled tobramycin (TOBI): a review of its use in the management of *Pseudomonas aeruginosa* infections in patients with cystic fibrosis Drugs 2003 63 22 2501 2520 10.2165/00003495-200363220-00015 14609360

[B34] Sudfeld CR Dasenbrook EC Merz WG Carroll KC Boyle MP Prevalence and risk factors for recovery of filamentous fungi in individuals with cystic fibrosis J Cyst Fibros 2010 9 2 110 116 10.1016/j.jcf.2009.11.010 20045384 PMC4113956

[B35] Kerr J Inhibition of fungal growth by *Pseudomonas aeruginosa* and *Pseudomonas cepacia* isolated from patients with cystic fibrosis J Infect 1994 28 305 310 10.1016/S0163-4453(94)91943-7 7522262

[B36] Mastella G Rainisio M Harms HK Hodson ME Koch C Navarro J Strandvik B McKenzie SG Allergic bronchopulmonary aspergillosis in cystic fibrosis. A European epidemiological study. Epidemiologic Registry of Cystic Fibrosis Eur Respir J 2000 16 3 464 471 10.1034/j.1399-3003.2000.016003464.x 11028661

[B37] Krcmery V Jr Matejicka F Pichnova E Jurga L Sulcova M Kunova A West D Documented fungal infections after prophylaxis or therapy with wide spectrum antibiotics: relationship between certain fungal pathogens and particular antimicrobials? J Chemother 1999 11 5 385 390 10.1179/joc.1999.11.5.385 10632385

[B38] Hebert C Villaran R Tolentino J Best L Boonlayangoor S Pitrak D Lin M Weber SG Prior antimicrobial exposure and the risk for bloodstream infection with fluconazole-non-susceptible *Candida* strains Scand J Infect Dis 2010 42 6–7 506 509 20370357 10.3109/00365541003699631

[B39] Wey SB Mori M Pfaller MA Woolson RF Wenzel RP Risk factors for hospital-acquired candidemia. A matched case–control study Arch Intern Med 1989 149 10 2349 2353 10.1001/archinte.1989.00390100145030 2802900

[B40] Charles PE Dalle F Aube H Doise JM Quenot JP Aho LS Chavanet P Blettery B *Candida* spp. colonization significance in critically ill medical patients: a prospective study Intensive Care Med 2005 31 3 393 400 10.1007/s00134-005-2571-y 15711782

[B41] Soysa NS Samaranayake LP Ellepola AN Antimicrobials as a contributory factor in oral candidosis–a brief overview Oral Dis 2008 14 2 138 143 10.1111/j.1601-0825.2006.01357.x 18302673

[B42] Xu J Schwartz K Bartoces M Monsur J Severson RK Sobel JD Effect of antibiotics on vulvovaginal candidiasis: a MetroNet study J Am Board Fam Med 2008 21 4 261 268 10.3122/jabfm.2008.04.070169 18612052

[B43] Samonis G Anastassiadou H Dassiou M Tselentis Y Bodey GP Effects of broad-spectrum antibiotics on colonization of gastrointestinal tracts of mice by *Candida albicans* Antimicrob Agents Chemother 1994 38 3 602 603 10.1128/AAC.38.3.602 8203861 PMC284504

[B44] Samonis G Gikas A Toloudis P Maraki S Vrentzos G Tselentis Y Tsaparas N Bodey G Prospective study of the impact of broad-spectrum antibiotics on the yeast flora of the human gut Eur J Clin Microbiol Infect Dis 1994 13 8 665 667 10.1007/BF01973996 7813500

[B45] Giuliano M Barza M Jacobus NV Gorbach SL Effect of broad-spectrum parenteral antibiotics on composition of intestinal microflora of humans Antimicrob Agents Chemother 1987 31 2 202 206 10.1128/AAC.31.2.202 3566249 PMC174692

[B46] Leaw SN Chang HC Sun HF Barton R Bouchara JP Chang TC Identification of medically important yeast species by sequence analysis of the internal transcribed spacer regions J Clin Microbiol 2006 44 3 693 699 10.1128/JCM.44.3.693-699.2006 16517841 PMC1393093

[B47] Pryce TM Palladino S Kay ID Coombs GW Rapid identification of fungi by sequencing the ITS1 and ITS2 regions using an automated capillary electrophoresis system Med Mycol 2003 41 5 369 381 10.1080/13693780310001600435 14653513

[B48] Ghannoum MA Jurevic RJ Mukherjee PK Cui F Sikaroodi M Naqvi A Gillevet PM Characterization of the oral fungal microbiome (mycobiome) in healthy individuals PLoS Pathog 2010 6 1 e1000713 10.1371/journal.ppat.1000713 20072605 PMC2795202

[B49] Ferrer C Colom F Frases S Mulet E Abad JL Alio JL Detection and identification of fungal pathogens by PCR and by ITS2 and 5.8S ribosomal DNA typing in ocular infections J Clin Microbiol 2001 39 8 2873 2879 10.1128/JCM.39.8.2873-2879.2001 11474006 PMC88253

[B50] Borman AM Linton CJ Miles SJ Johnson EM Molecular identification of pathogenic fungi J Antimicrob Chemother 2008 61 Suppl 1 i7 i12 18063605 10.1093/jac/dkm425

[B51] Spiess B Seifarth W Hummel M Frank O Fabarius A Zheng C Morz H Hehlmann R Buchheidt D DNA microarray-based detection and identification of fungal pathogens in clinical samples from neutropenic patients J Clin Microbiol 2007 45 11 3743 3753 10.1128/JCM.00942-07 17715373 PMC2168469

[B52] Kittelmann S Naylor GE Koolaard JP Janssen PH A proposed taxonomy of anaerobic fungi (class *neocallimastigomycetes*) suitable for large-scale sequence-based community structure analysis PLoS One 2012 7 5 e36866 10.1371/journal.pone.0036866 22615827 PMC3353986

[B53] Huse SM Mark DB Welch A Voorhis A Shipunova HG Morrison AM Eren Sogin ML VAMPS: a website for visualization and analysis of microbial population structures BMC Bioinformatics 2014 15 1 41 10.1186/1471-2105-15-41 24499292 PMC3922339

[B54] Huse SM Dethlefsen L Huber JA Mark D Welch DA Relman Sogin ML Exploring microbial diversity and taxonomy using SSU rRNA hypervariable tag sequencing PLoS Genet 2008 4 11 e1000255 10.1371/journal.pgen.1000255 19023400 PMC2577301

[B55] Huse SM Welch DM Morrison HG Sogin ML Ironing out the wrinkles in the rare biosphere through improved OTU clustering Environ Microbiol 2010 12 7 1889 1898 10.1111/j.1462-2920.2010.02193.x 20236171 PMC2909393

[B56] Huber JA Mark Welch DB Morrison HG Huse SM Neal PR Butterfield DA Sogin ML Microbial population structures in the deep marine biosphere Science 2007 318 5847 97 100 10.1126/science.1146689 17916733

[B57] Madan JC Koestler DC Stanton BA Davidson L Moulton LA Housman ML Moore JH Guill MF Morrison HG Sogin ML Hampton TH Karagas MR Palumbo PE Foster JA Hibberd PL O’Toole GA Serial analysis of the gut and respiratory microbiome in cystic fibrosis in infancy: interaction between intestinal and respiratory tracts and impact of nutritional exposures MBio 2012 3 4 e00251 12 22911969 10.1128/mBio.00251-12 PMC3428694

[B58] Koljalg U Nilsson RH Abarenkov K Tedersoo L Taylor AF Bahram M Bates ST Bruns TD Bengtsson-Palme J Callaghan TM Douglas B Drenkhan T Eberhardt U Duenas M Grebenc T Griffith GW Hartmann M Kirk PM Kohout P Larsson E Lindahl BD Lucking R Martin MP Matheny PB Nguyen NH Niskanen T Oja J Peay KG Peintner U Peterson M Towards a unified paradigm for sequence-based identification of fungi Mol Ecol 2013 22 21 5271 5277 10.1111/mec.12481 24112409

[B59] Bruns T White T Taylor J Fungal molecular systematics Annu Rev Ecol Syst 1991 22 525 564 10.1146/annurev.es.22.110191.002521

[B60] Yao C Frederiksen RA Magill CW Length heterogeneity in ITS 2 and the methylation status of CCGG and GCGC sites in the rRNA genes of the genus *Peronosclerospora* Curr Genet 1992 22 5 415 420 10.1007/BF00352443 1423729

[B61] Longo AV Rodriguez D Da Silva D Leite LF Toledo C Mendoza A Burrowes PA Zamudio KR ITS1 copy number varies among Batrachochytrium dendrobatidis strains: implications for qPCR estimates of infection intensity from field-collected amphibian skin swabs PLoS One 2013 8 3 e59499 10.1371/journal.pone.0059499 23555682 PMC3605245

[B62] Nilsson RH Kristiansson E Ryberg M Hallenberg N Larsson KH Intraspecific ITS variability in the kingdom fungi as expressed in the international sequence databases and its implications for molecular species identification Evol Bioinform Online 2008 4 193 201 19204817 10.4137/ebo.s653 PMC2614188

[B63] Bittar F Rolain JM Detection and accurate identification of new or emerging bacteria in cystic fibrosis patients Clin Microbiol Infect 2010 16 7 809 820 10.1111/j.1469-0691.2010.03236.x 20880410

[B64] Sibley CD Rabin H Surette MG Cystic fibrosis: a polymicrobial infectious disease Future Microbiol 2006 1 1 53 61 10.2217/17460913.1.1.53 17661685

[B65] Zemanick ET Harris JK Wagner BD Robertson CE Sagel SD Stevens MJ Accurso FJ Laguna TA Inflammation and airway microbiota during cystic fibrosis pulmonary exacerbations PLoS One 2013 8 4 e62917 10.1371/journal.pone.0062917 23646159 PMC3639911

[B66] Hirschi S Letscher-Bru V Pottecher J Lannes B Jeung MY Degot T Santelmo N Sabou AM Herbrecht R Kessler R Disseminated *Trichosporon mycotoxinivorans*, *Aspergillus fumigatus*, and *Scedosporium apiospermum* coinfection after lung and liver transplantation in a cystic fibrosis patient J Clin Microbiol 2012 50 12 4168 4170 10.1128/JCM.01928-12 23035187 PMC3503015

[B67] Kothavade RJ Kura MM Valand AG Panthaki MH *Candida tropicalis*: its prevalence, pathogenicity and increasing resistance to fluconazole J Med Microbiol 2010 59 Pt 8 873 880 20413622 10.1099/jmm.0.013227-0

[B68] Davies AN Brailsford S Broadley K Beighton D Oral yeast carriage in patients with advanced cancer Oral Microbiol Immunol 2002 17 2 79 84 10.1046/j.0902-0055.2001.00095.x 11929553

[B69] Xu J Mitchell TG Geographical differences in human oral yeast flora Clin Infect Dis 2003 36 2 221 224 10.1086/345672 12522756

[B70] Silva S Negri M Henriques M Oliveira R Williams DW Azeredo J *Candida glabrata*, *Candida parapsilosis* and *Candida tropicalis*: biology, epidemiology, pathogenicity and antifungal resistance FEMS Microbiol Rev 2012 36 2 288 305 10.1111/j.1574-6976.2011.00278.x 21569057

[B71] Mounier J Gouello A Keravec M Le Gal S Pacini G Debaets S Nevez G Rault G Barbier G Hery-Arnaud G Use of denaturing high-performance liquid chromatography (DHPLC) to characterize the bacterial and fungal airway microbiota of cystic fibrosis patients J Microbiol 2014 52 4 307 314 10.1007/s12275-014-3425-5 24535743

[B72] Findley K Oh J Yang J Conlan S Deming C Meyer JA Schoenfeld D Nomicos E Park M Kong HH Segre JA Topographic diversity of fungal and bacterial communities in human skin Nature 2013 498 7454 367 370 10.1038/nature12171 23698366 PMC3711185

[B73] Hauser PM Bernard T Greub G Jaton K Pagni M Hafen GM Microbiota present in cystic fibrosis lungs as revealed by whole genome sequencing PLoS One 2014 9 3 e90934 10.1371/journal.pone.0090934 24599149 PMC3944733

[B74] Charlson ES Diamond JM Bittinger K Fitzgerald AS Yadav A Haas AR Bushman FD Collman RG Lung-enriched organisms and aberrant bacterial and fungal respiratory microbiota after lung transplant Am J Respir Crit Care Med 2012 186 6 536 545 10.1164/rccm.201204-0693OC 22798321 PMC3480531

[B75] Hogan DA Vik A Kolter R A *Pseudomonas aeruginosa* quorum-sensing molecule influences *Candida albicans* morphology Mol Microbiol 2004 54 5 1212 1223 10.1111/j.1365-2958.2004.04349.x 15554963

[B76] Morales DK Grahl N Okegbe C Dietrich LE Jacobs NJ Hogan DA Control of *Candida albicans* metabolism and biofilm formation by *Pseudomonas aeruginosa* phenazines MBio 2013 4 1 e00526 12 23362320 10.1128/mBio.00526-12 PMC3560528

[B77] Hogan DA Kolter R *Pseudomonas-Candida* interactions: an ecological role for virulence factors Science 2002 296 5576 2229 2232 10.1126/science.1070784 12077418

[B78] Gibson J Sood A Hogan DA *Pseudomonas aeruginosa-Candida albicans* interactions: localization and fungal toxicity of a phenazine derivative Appl Environ Microbiol 2009 75 2 504 513 10.1128/AEM.01037-08 19011064 PMC2620721

[B79] Lindsay AK Morales DK Liu Z Grahl N Zhang A Willger SD Myers LC Hogan DA Analysis of *Candida albicans* mutants defective in the Cdk8 module of mediator reveal links between metabolism and biofilm formation PLoS Genet 2014 10 10 e1004567 10.1371/journal.pgen.1004567 25275466 PMC4183431

[B80] Cugini C Morales DK Hogan DA *Candida albicans*-produced farnesol stimulates *Pseudomonas* quinolone signal production in LasR-defective *Pseudomonas aeruginosa* strains Microbiology 2010 156 Pt 10 3096 3107 20656785 10.1099/mic.0.037911-0 PMC3068698

[B81] McAlester G O’Gara F Morrissey JP Signal-mediated interactions between *Pseudomonas aeruginosa* and *Candida albicans* J Med Microbiol 2008 57 Pt 5 563 569 18436588 10.1099/jmm.0.47705-0

[B82] Samonis G Gikas A Anaissie EJ Vrenzos G Maraki S Tselentis Y Bodey GP Prospective evaluation of effects of broad-spectrum antibiotics on gastrointestinal yeast colonization of humans Antimicrob Agents Chemother 1993 37 1 51 53 10.1128/AAC.37.1.51 8431017 PMC187603

[B83] Mulligan ME Citron DM McNamara BT Finegold SM Impact of cefoperazone therapy on fecal flora Antimicrob Agents Chemother 1982 22 2 226 230 10.1128/AAC.22.2.226 6927284 PMC183716

[B84] Abbott J Clinical and microscopic diagnosis of vaginal yeast infection: a prospective analysis Ann Emerg Med 1995 25 5 587 591 10.1016/S0196-0644(95)70168-0 7741332

[B85] Oksala E Factors predisposing to oral yeast infections Acta Odontol Scand 1990 48 1 71 74 10.3109/00016359009012736 2181813

[B86] Leyden JJ Marples RR Ecologic principles and antibiotic therapy in chronic dermatoses Arch Dermatol 1973 107 2 208 211 10.1001/archderm.1973.01620170020006 4265456

[B87] Ben-Ami R Olshtain-Pops K Krieger M Oren I Bishara J Dan M Wiener-Well Y Weinberger M Zimhony O Chowers M Weber G Potasman I Chazan B Kassis I Shalit I Block C Keller N Kontoyiannis DP Giladi M Antibiotic exposure as a risk factor for fluconazole-resistant *Candida* bloodstream infection Antimicrob Agents Chemother 2012 56 5 2518 2523 10.1128/AAC.05947-11 22314534 PMC3346668

[B88] Bow EJ Louie TJ Changes in endogenous microflora among febrile granulocytopenic patients receiving empiric antibiotic therapy: implications for fungal superinfection CMAJ 1987 137 5 397 403 3304600 PMC1492778

[B89] Dollive S Chen YY Grunberg S Bittinger K Hoffmann C Vandivier L Cuff C Lewis JD Wu GD Bushman FD Fungi of the murine gut: episodic variation and proliferation during antibiotic treatment PLoS One 2013 8 8 e71806 10.1371/journal.pone.0071806 23977147 PMC3747063

[B90] Rogers GB Cuthbertson L Hoffman LR Wing PA Pope C Hooftman DA Lilley AK Oliver A Carroll MP Bruce KD van der Gast CJ Reducing bias in bacterial community analysis of lower respiratory infections ISME J 2013 7 4 697 706 10.1038/ismej.2012.145 23190732 PMC3603400

[B91] Young VB Raffals LH Huse SM Vital M Dai D Schloss PD Brulc JM Antonopoulos DA Arrieta RL Kwon JH Reddy KG Hubert NA Grim SL Vineis JH Dalal S Morrison HG Eren AM Meyer F Schmidt TM Tiedje JM Chang EB Sogin ML Multiphasic analysis of the temporal development of the distal gut microbiota in patients following ileal pouch anal anastomosis Microbiome 2013 1 1 9 10.1186/2049-2618-1-9 24451366 PMC3971607

[B92] Huse SM Huber JA Morrison HG Sogin ML Welch DM Accuracy and quality of massively parallel DNA pyrosequencing Genome Biol 2007 8 7 R143 10.1186/gb-2007-8-7-r143 17659080 PMC2323236

[B93] Edgar RC Haas BJ Clemente JC Quince C Knight R UCHIME improves sensitivity and speed of chimera detection Bioinformatics 2011 27 16 2194 2200 10.1093/bioinformatics/btr381 21700674 PMC3150044

[B94] Huse SM Young VB Morrison HG Antonopoulos DA Kwon J Dalal S Arrieta R Hubert NA Shen L Vineis JH Koval JC Sogin ML Chang EB Raffals LE Comparison of brush and biopsy sampling methods of the ileal pouch for assessment of mucosa-associated microbiota of human subjects Microbiome 2014 2 1 5 10.1186/2049-2618-2-5 24529162 PMC3931571

[B95] Eren AM Vineis JH Morrison HG Sogin ML A filtering method to generate high quality short reads using illumina paired-end technology PLoS One 2013 8 6 e66643 10.1371/journal.pone.0066643 23799126 PMC3684618

[B96] Quast C Pruesse E Yilmaz P Gerken J Schweer T Yarza P Peplies J Glockner FO The SILVA ribosomal RNA gene database project: improved data processing and web-based tools Nucleic Acids Res 2013 41 Database issue D590 D596 23193283 10.1093/nar/gks1219 PMC3531112

[B97] Edgar RC Search and clustering orders of magnitude faster than BLAST Bioinformatics 2010 26 19 2460 2461 10.1093/bioinformatics/btq461 20709691

[B98] Caporaso JG Bittinger K Bushman FD DeSantis TZ Andersen GL Knight R PyNAST: a flexible tool for aligning sequences to a template alignment Bioinformatics 2010 26 2 266 267 10.1093/bioinformatics/btp636 19914921 PMC2804299

[B99] Jacobs J Individual Based Rarefaction using R-package 2011

[B100] Liu CM Kachur S Dwan MG Abraham AG Aziz M Hsueh PR Huang YT Busch JD Lamit LJ Gehring CA Keim P Price LB FungiQuant: a broad-coverage fungal quantitative real-time PCR assay BMC Microbiol 2012 12 255 10.1186/1471-2180-12-255 23136846 PMC3565980

[B101] Horz HP Vianna ME Gomes BP Conrads G Evaluation of universal probes and primer sets for assessing total bacterial load in clinical samples: general implications and practical use in endodontic antimicrobial therapy J Clin Microbiol 2005 43 10 5332 5337 10.1128/JCM.43.10.5332-5337.2005 16208011 PMC1248440

[B102] Maeda H Fujimoto C Haruki Y Maeda T Kokeguchi S Petelin M Arai H Tanimoto I Nishimura F Takashiba S Quantitative real-time PCR using TaqMan and SYBR Green for *Actinobacillus actinomycetemcomitans*, *Porphyromonas gingivalis*, *Prevotella intermedia*, *tetQ* gene and total bacteria FEMS Immunol Med Microbiol 2003 39 1 81 86 10.1016/S0928-8244(03)00224-4 14557000

[B103] Goslee SC Urban DL The ecodist package for dissimilarity-based analysis of ecological data J Stat Softw 2007 22 1 19

[B104] Dixon P VEGAN, a package of R functions for community ecology J Veg Sci 2003 14 6 927 930 10.1111/j.1654-1103.2003.tb02228.x

