# Supplementary material for: Characterization and quantification of the fungal microbiome in serial samples from individuals with cystic fibrosis
Source: Microbiome. 2014 Nov 3;2:40. doi: 10.1186/2049-2618-2-40 (PMC4236224; doi:10.1186/2049-2618-2-40)
Supplement: Additional file 1: Table S1 — Microbiological results from samples collected at the time of admission for treatment of exacerbation and antibacterial drugs administered. [file 2049-2618-2-40-S1.doc]

**Willger et al. 2014**

**Supplemental Tables**.

**Table S1**. Microbiological results from samples collected at the time of admission for treatment of exacerbation and antibacterial drugs administered.

| **Subject #** | **Sputum culture results** | |  |
| --- | --- | --- | --- |
|  | **Fungi** | **Bacteria** | **Antibacterial therapy** |
| 1 | *Candida parapsilosis* | Mucoid *Pa,*  *S. aureus* | Tobramycin, Meropenem |
| 2 | *Candida dubliniensis* | *MRSA,*  *P. fluorescens,*  *P. putida* | Tobramycin, Ceftazadime, Linezolid |
| 3 | *Candida parapsilosis* | Mucoid *Pa* | Tobramycin, Ceftazadime |
| 6 | - | *Pa* | Tobramycin, Vancomycin, Doripenem |
| 8 | - | Mucoid *Pa* | Tobramycin, Vancomycin, Doripenem |
| 9 | - | *S. aureus*, *Pa* and Mucoid *Pa* | Tobramycin, Doripenem |

*Pa*, *Pseudomonas aeruginosa*; MRSA, Methicillin-resistant *Staphylococcus aureus*;

-,none reported.
