# Supplementary figures and images for: Characterization and quantification of the fungal microbiome in serial samples from individuals with cystic fibrosis
Source: Microbiome. 2014 Nov 3;2:40. doi: 10.1186/2049-2618-2-40 (PMC4236224; doi:10.1186/2049-2618-2-40)

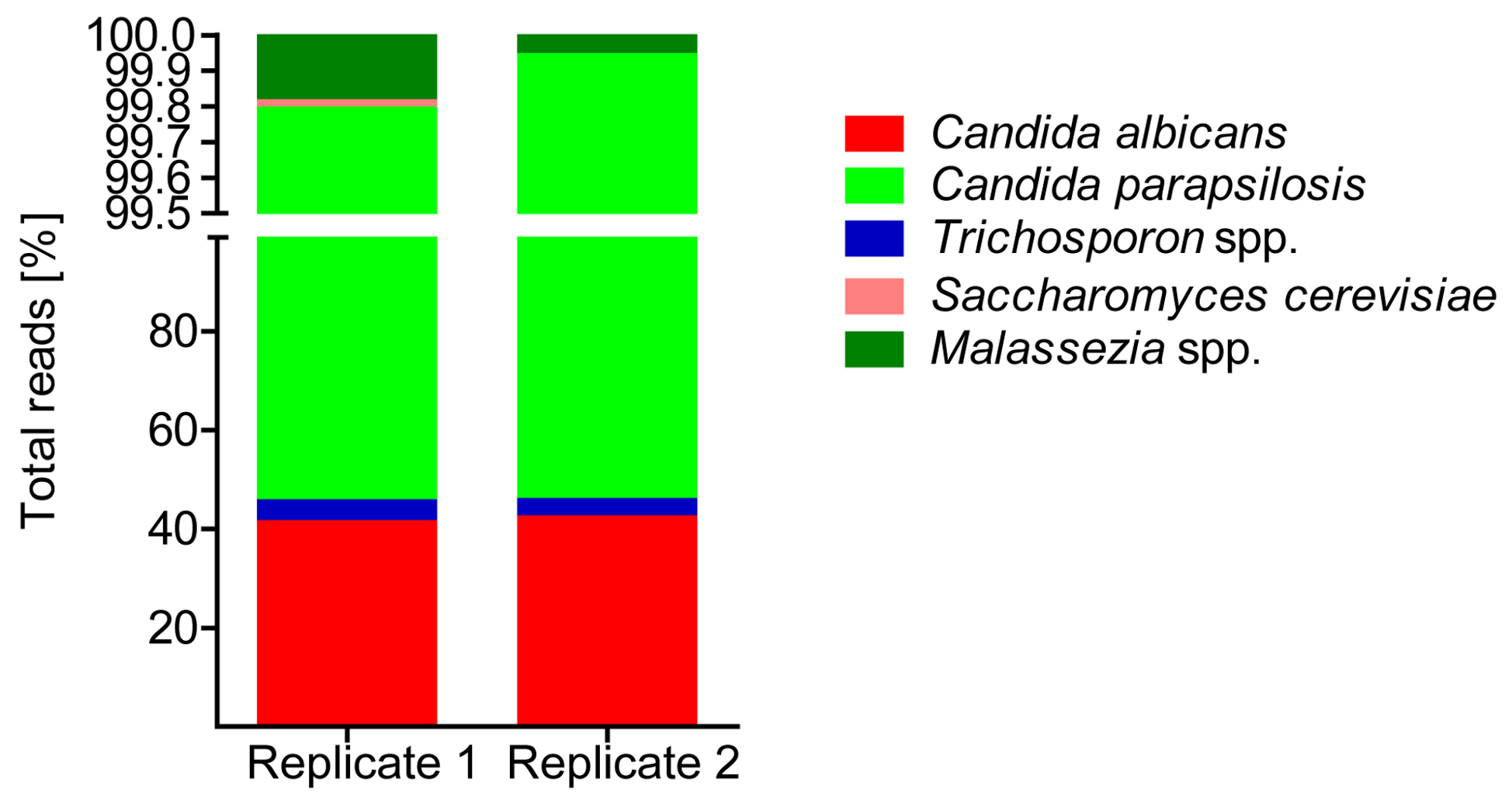

Supplement: Additional file 2: Figure S1 — Analysis of amplification and sequencing method reproducibility. DNA from a sputum sample was divided, amplified, sequenced, and analyzed in parallel to evaluate our methods. [file 2049-2618-2-40-S2.tiff]

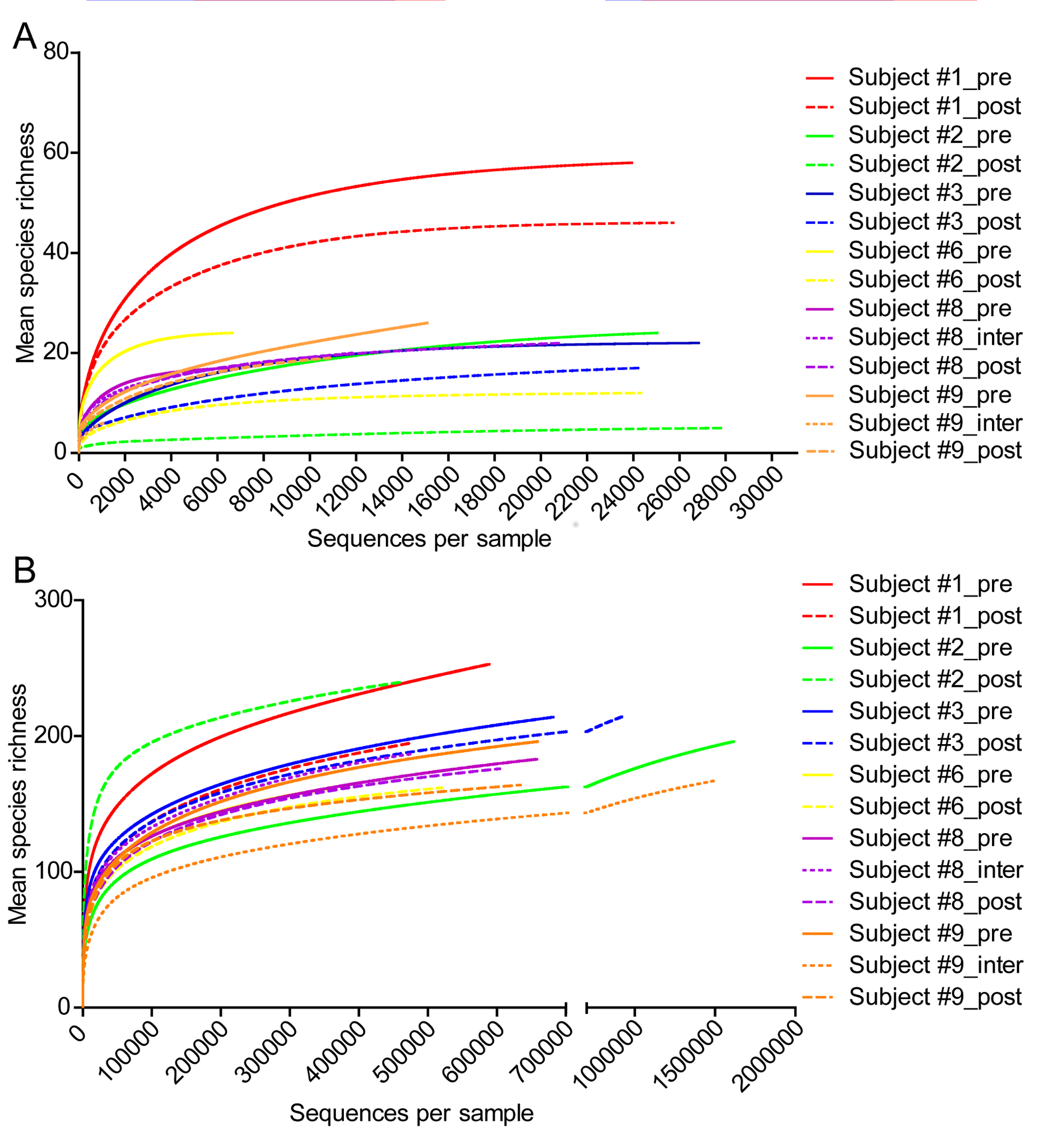

Supplement: Additional file 4: Figure S2 — Rarefaction analysis of the mycobiome and microbiome. Rarefaction curves to determine the completeness of deep sequencing of the mycobiome (A) and the microbiome (B). Repeated samples of species subsets were used to evaluate whether further sampling would likely yield additional species, as indicated by whether the curve has not yet reached a plateau. The y-axis indicates the number of species detected and the x-axis the number of sequences analyzed per sample. [file 2049-2618-2-40-S4.tiff]

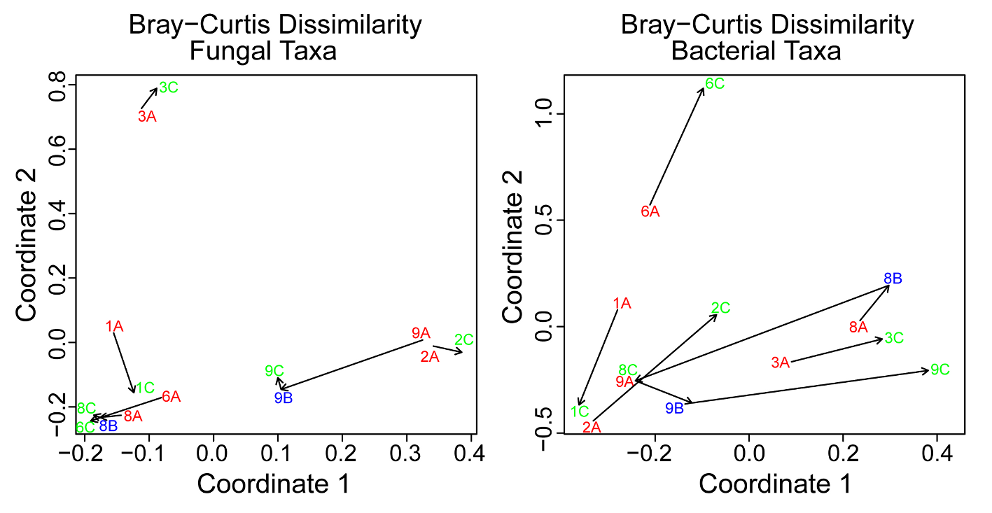

Supplement: Additional file 8: Figure S3 — Principal component analysis based on Bray-Curtis dissimilarity. Bray-Curtis dissimilarity and principal coordinate analysis were used to measure and represent taxonomic relatedness between classes of samples in the mycobiome (fungal taxa) and the microbiome (bacterial taxa) samples. Pre-treatment samples are colored in red and are labeled A, intermediate samples collected during treatment are colored blue and are labeled B, and post-treatment samples are colored green and are labeled C. The numbers indicate the subject number. [file 2049-2618-2-40-S8.tiff]

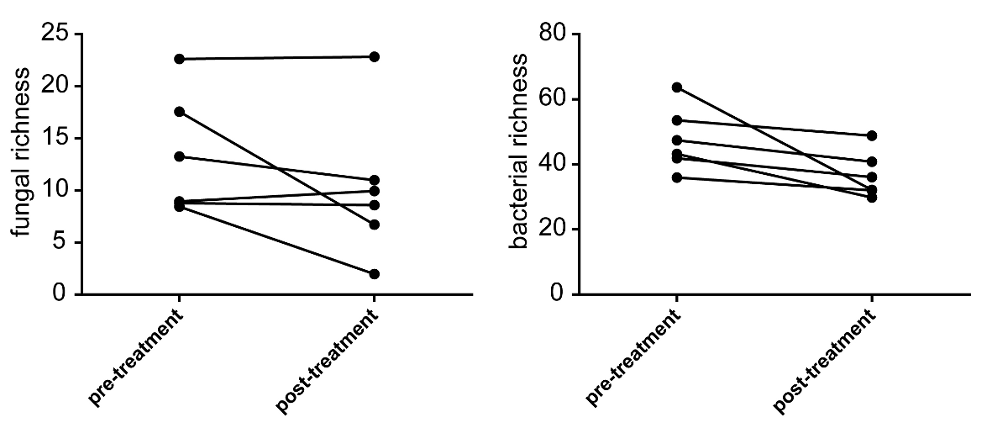

Supplement: Additional file 10: Figure S4 — Comparison of the normalized richness of the mycobiome and microbiome. Comparison of the normalized mean fungal and bacterial richness of pre-treatment and post-treatment samples. The normalized mean fungal richness shows no significant difference between treatments (P >0.1) but the normalized mean bacterial richness decreases upon antibacterial treatment (P <0.05) (Wilcoxon matched-pairs signed rank test). [file 2049-2618-2-40-S10.tiff]

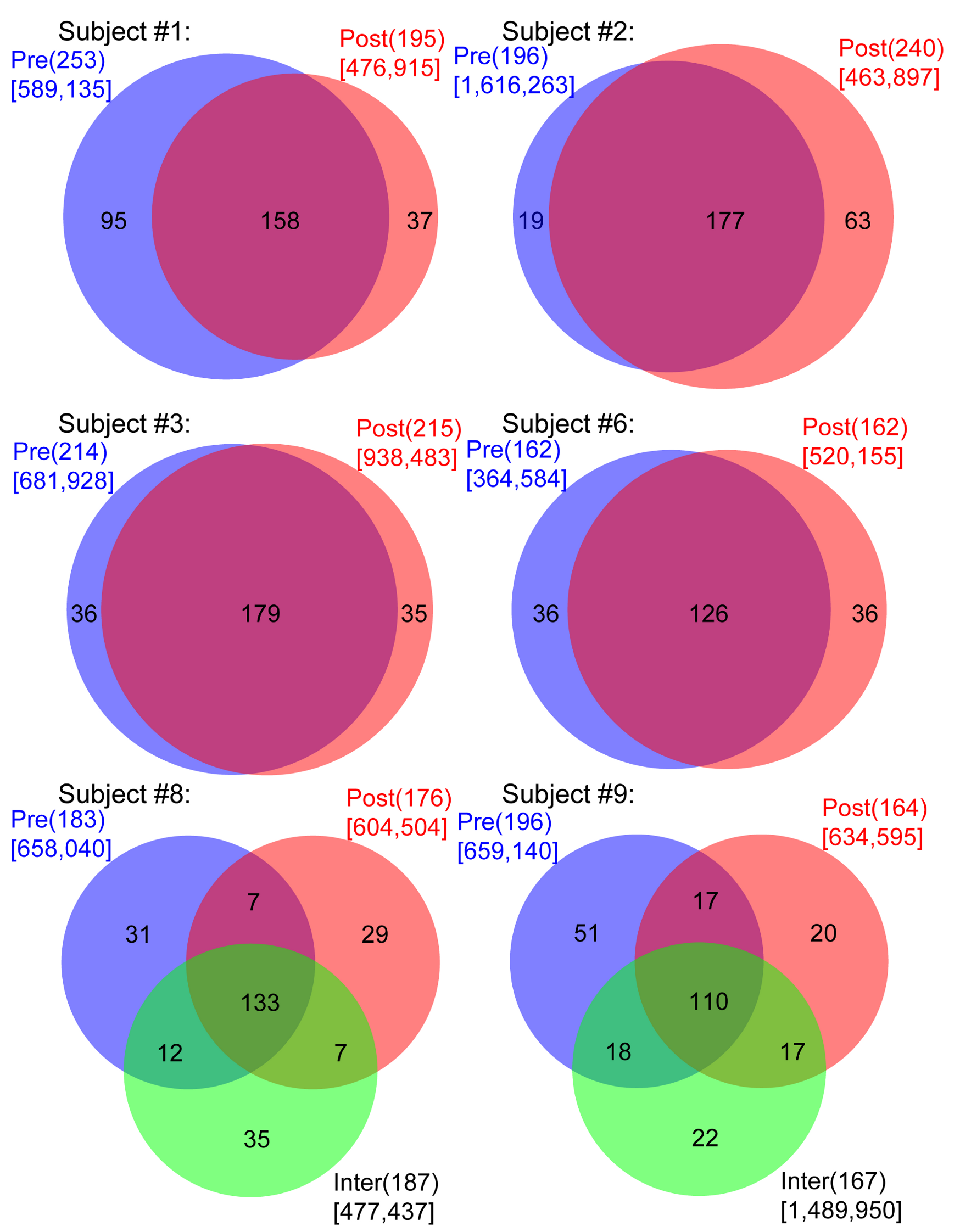

Supplement: Additional file 12: Figure S5 — Bacterial communities within a subject before, during, and after treatment. Distribution of bacterial taxa detected in CF subjects either immediately after an exacerbation and before starting an antibacterial therapy (pre), approximately 2 weeks afterwards (post) or while hospitalized (inter) presented in Euler diagrams for the pre- and post-samples (subjects #1–6) and Venn diagrams for series with three samples (subjects #8 and 9). The numbers in parenthesis describe the total number of taxa detected in a sample; the numbers in the circles represent either the unique number of taxa in a sample or the number of shared taxa in the overlap regions. [file 2049-2618-2-40-S12.tiff]

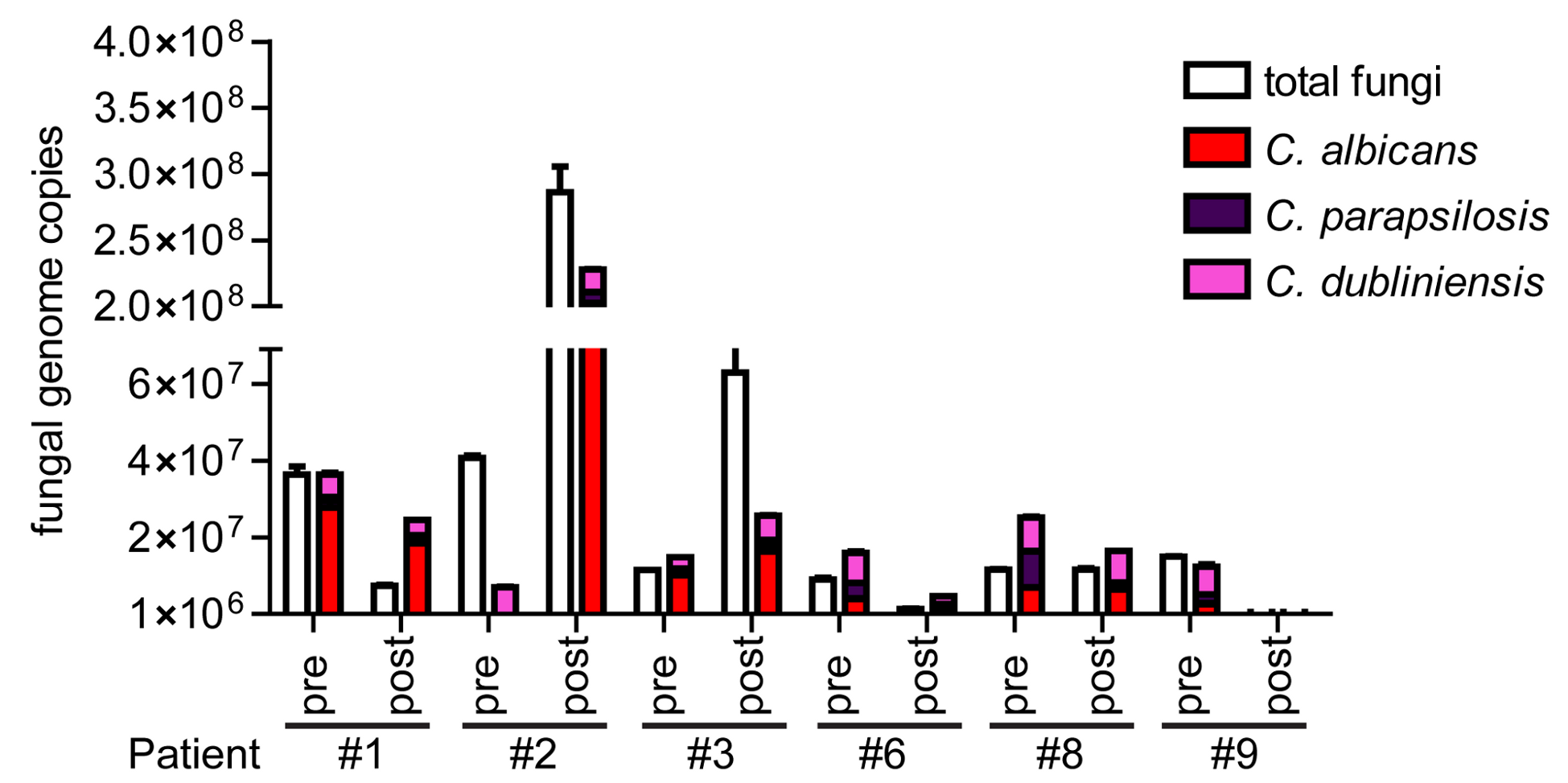

Supplement: Additional file 14: Figure S6 — Quantification of total fungi and specific Candida species in sputum of CF subjects. Quantification of the fungal burden of sputum samples from CF subjects. Fungal burden was determined by amplifying the 18S rDNA locus by qPCR. For Candida species-specific quantification, we used the genes CAWG_05066 (C. albicans), CPAR2_301290 (C. parapsilosis), and Cd36_16280 (C. dubliniensis). Fungal genome copy number was determined by comparison to a standard curve generated using DNA isolated from pure cultures of Candida albicans as described in the methods. [file 2049-2618-2-40-S14.tiff]
