# Supplementary material for: Characterization and quantification of the fungal microbiome in serial samples from individuals with cystic fibrosis
Source: Microbiome. 2014 Nov 3;2:40. doi: 10.1186/2049-2618-2-40 (PMC4236224; doi:10.1186/2049-2618-2-40)
Supplement: Additional file 3: Table S3 — Total number of reads used in the mycobiome (fungi) and the bacterial microbiome analyses. [file 2049-2618-2-40-S3.doc]

**Table S3. Number of reads used in VAMPS analysis of the mycobiome (fungi) and the microbiome (bacteria**)

| **Sample ID** | **VAMPS ID** | **fungi** | **VAMPS ID** | **bacteria** |
| --- | --- | --- | --- | --- |
| Subject #1_pre | Subject #1_pre_ITS | 22,376 | Subject #1_pre_16S_V6 | 589,135 |
| Subject #1_post | Subject #1_post_ITS | 24,260 | Subject #1_post_16S_V6 | 476,915 |
| Subject #2_pre | Subject #2_pre_ITS | 23,983 | Subject #2_pre_16S_V6 | 1,616,263 |
| Subject #2_post | Subject #2_post_ITS | 27,077 | Subject #2_post_16S_V6 | 463,897 |
| Subject #3_pre | Subject #3_pre_ITS | 25,785 | Subject #3_pre_16S_V6 | 681,928 |
| Subject #3_post | Subject #3_post_ITS | 23,027 | Subject #3_post_16S_V6 | 938,483 |
| Subject #6_pre | Subject #6_pre_ITS | 10,878 | Subject #6_pre_16S_V6 | 364,584 |
| Subject #6_post | Subject #6_post_ITS | 37,684 | Subject #6_post_16S_V6 | 520,155 |
| Subject #8_pre | Subject #8_pre_ITS | 10,822 | Subject #8_pre_16S_V6 | 658,040 |
| Subject #8_inter | Subject #8_inter_ITS | 18,599 | Subject #8_inter_16S_V6 | 477,437 |
| Subject #8_post | Subject #8_post_ITS | 32,919 | Subject #8_post_16S_V6 | 604,504 |
| Subject #9_pre | Subject #9_pre_ITS | 23,910 | Subject #9_pre_16S_V6 | 659,140 |
| Subject #9_inter | Subject #9_inter_ITS | 1,672 | Subject #9_inter_16S_V6 | 1,489,950 |
| Subject #9_post | Subject #9_post_ITS | 17,080 | Subject #9_post_16S_V6 | 634,595 |
