# Supplementary material for: Characterization and quantification of the fungal microbiome in serial samples from individuals with cystic fibrosis
Source: Microbiome. 2014 Nov 3;2:40. doi: 10.1186/2049-2618-2-40 (PMC4236224; doi:10.1186/2049-2618-2-40)
Supplement: Additional file 7: Table S2 — Primer list. [file 2049-2618-2-40-S7.doc]

**Table S2**. Primer list

| **Primer Name** | **Primer Sequence (5’-3’)** |
| --- | --- |
| ITS1_F | GTA AAA GTC GTA ACA AGG TTT C |
| ITS1_R | GTT CAA AGA YTC GAT GAT TCA C |
| ITS1_mblb F with adapter | CGTATCGCCTCCCTCGCGCCATCAGGTAAAAGTCGTAACAAGGTTTC |
| ITS1_mblb_Bar144L | CTATGCGCCTTGCCAGCCCGCTCATATCAACGTTCAAAGAYTCGATGATTCAC |
| ITS1_mblb_Bar146L | CTATGCGCCTTGCCAGCCCGCTCATATTGACGTTCAAAGAYTCGATGATTCAC |
| ITS1_mblb_Bar166L | CTATGCGCCTTGCCAGCCCGCTCATGACGACGTTCAAAGAYTCGATGATTCAC |
| ITS1_mblb_Bar213L | CTATGCGCCTTGCCAGCCCGCTCATCACCTCGTTCAAAGAYTCGATGATTCAC |
| ITS1_mblb_Bar560L | CTATGCGCCTTGCCAGCCCGCTCAAGACCTCGTTCAAAGAYTCGATGATTCAC |
| ITS1_mblb_Bar575L | CTATGCGCCTTGCCAGCCCGCTCAAGGCGGCGTTCAAAGAYTCGATGATTCAC |
| ITS1_mblb_Bar613L | CTATGCGCCTTGCCAGCCCGCTCAACAAGGCGTTCAAAGAYTCGATGATTCAC |
| ITS1_mblb_Bar741L | CTATGCGCCTTGCCAGCCCGCTCAATACCACGTTCAAAGAYTCGATGATTCAC |
| ITS1_mblb_Bar1149L | CTATGCGCCTTGCCAGCCCGCTCAAAGCCGCGTTCAAAGAYTCGATGATTCAC |
| ITS1_mblb_Bar1172L | CTATGCGCCTTGCCAGCCCGCTCAAAGGCACGTTCAAAGAYTCGATGATTCAC |
| ITS1_mblb_Bar1273L | CTATGCGCCTTGCCAGCCCGCTCAAACCAGCGTTCAAAGAYTCGATGATTCAC |
| FungiQuant_RT_F | GSWCTATCCCCAKCACGA |
| FungiQuant_RT_R | GGRAAACTCACCAGGTCCAG |
| *C. albicans* specific_F | ACTTCTGTAAGAGTGCTGGTTC |
| *C. albicans* specific_R | TGTCGTAATCAAACTCGGTAGC |
| *C. parapsilosis* specific_F | GCATGCCAGGAGAGTGTAAA |
| *C. parapsilosis* specific_R | GGTGCAATTCCAGACGTATCA |
| *C. dubliniensis* specific_F | TGTGGAATTTGGTTTCCCATTATC |
| *C. dubliniensis* specific_R | GCGTTGAAATCTGAAGGAAGAAG |
| *C. tropicalis* specific_F | GCTGGTACTAAACTCGGTCATAA |
| *C. tropicalis* specific_R | CGCAATGGGAAACAACCATAATA |
| total bacteria_F | GTGSTGCAYGGYTGTCGTCA |
| total bacteria_R | ACGTCRTCCMCACCTTCCTC |
| ITS1F-N4TCAGC | AATGATACGGCGACCACCGAGATCTACACTCTTTCCCTACACGACGCTCTTCCGATCTNNNNTCAGCGTAAAAGTCGTAACAAGGTTTC |
| ITS1R-idx37 | CAAGCAGAAGACGGCATACGAGATCACTACGTGACTGGAGTTCAGACGTGTGCTCTTCCGATCTGTTCAAAGAYTCGATGATTCAC |
| ITS1R-idx38 | CAAGCAGAAGACGGCATACGAGATTGCAGTGTGACTGGAGTTCAGACGTGTGCTCTTCCGATCTGTTCAAAGAYTCGATGATTCAC |
| ITS1R-idx39 | CAAGCAGAAGACGGCATACGAGATACCATAGTGACTGGAGTTCAGACGTGTGCTCTTCCGATCTGTTCAAAGAYTCGATGATTCAC |
| ITS1R-idx40 | CAAGCAGAAGACGGCATACGAGATTCGACAGTGACTGGAGTTCAGACGTGTGCTCTTCCGATCTGTTCAAAGAYTCGATGATTCAC |
| ITS1R-idx41 | CAAGCAGAAGACGGCATACGAGATGAACACGTGACTGGAGTTCAGACGTGTGCTCTTCCGATCTGTTCAAAGAYTCGATGATTCAC |
| ITS1R-idx42 | CAAGCAGAAGACGGCATACGAGATGAGCCAGTGACTGGAGTTCAGACGTGTGCTCTTCCGATCTGTTCAAAGAYTCGATGATTCAC |
| ITS1R-idx43 | CAAGCAGAAGACGGCATACGAGATTTGGGTGTGACTGGAGTTCAGACGTGTGCTCTTCCGATCTGTTCAAAGAYTCGATGATTCAC |
| ITS1R-idx44 | CAAGCAGAAGACGGCATACGAGATAAGGCGGTGACTGGAGTTCAGACGTGTGCTCTTCCGATCTGTTCAAAGAYTCGATGATTCAC |
| ITS1R-idx45 | CAAGCAGAAGACGGCATACGAGATTAATACGTGACTGGAGTTCAGACGTGTGCTCTTCCGATCTGTTCAAAGAYTCGATGATTCAC |
| ITS1R-idx46 | CAAGCAGAAGACGGCATACGAGATTCGGAAGTGACTGGAGTTCAGACGTGTGCTCTTCCGATCTGTTCAAAGAYTCGATGATTCAC |
| ITS1R-idx47 | CAAGCAGAAGACGGCATACGAGATTGTGAAGTGACTGGAGTTCAGACGTGTGCTCTTCCGATCTGTTCAAAGAYTCGATGATTCAC |
| ITS1R-idx48 | CAAGCAGAAGACGGCATACGAGATCATTCGGTGACTGGAGTTCAGACGTGTGCTCTTCCGATCTGTTCAAAGAYTCGATGATTCAC |
