# Supplementary material for: Characterization and quantification of the fungal microbiome in serial samples from individuals with cystic fibrosis
Source: Microbiome. 2014 Nov 3;2:40. doi: 10.1186/2049-2618-2-40 (PMC4236224; doi:10.1186/2049-2618-2-40)
Supplement: Additional file 11 — Mycobiome data analysis. [file 2049-2618-2-40-S11.docx]

**Supplemental Method**:

Mycobiome Data Analysis

High-quality ITS1 sequence datasets were combined to identify unique sequences and their frequency using the PivotTable tool in Excel. The file with the unique sequences is further processed by custom PERL scripts (Supplemental material) to remove all sequences shorter than 50 bp and convert the remaining unique sequences to FASTA format. We used the web-based workbench PlutoF (<http://plutof.ut.ee/>) to extract all ITS1 sequences from hosting the UNITE database (<http://unite.ut.ee/index.php>) to extract all ITS1 sequences from the file of unique sequences with the “ITS extractor”. These ITS1 sequences served as input to query the International Nucleotide Sequence Database (INSD) using the tool “massBLASTer” on the UNITE website. The analysis options chosen for the BLAST search were a regular output style with 1 alignment shown and algorithm parameters were set for similar sequences (fast).

We verified fungal species assignments and binned sequences classified as the same species. Finally, the taxonomic assignment of each unique sequence was integrated with its frequency to calculate the proportion of each fungal species present in each sample.
